# Gut Microbiome and Putative Resistome of Inca and Italian Nobility Mummies

**DOI:** 10.3390/genes8110310

**Published:** 2017-11-07

**Authors:** Tasha M. Santiago-Rodriguez, Gino Fornaciari, Stefania Luciani, Gary A. Toranzos, Isolina Marota, Valentina Giuffra, Raul J. Cano

**Affiliations:** 1 Center for Applications in Biotechnology, California Polytechnic State University, San Luis Obispo, CA 93407, USA; 2 Department of Biology, California Polytechnic State University, San Luis Obispo, CA 93407, USA; 3 ATCC-Center for Translational Microbiology, Institute for Life Science Entrepreneurship, Union, NJ 07083, USA; 4 Department of Translational Research on New Technologies in Medicine and Surgery, Division of Paleopathology, University of Pisa, 56126 Pisa, Italy; gino.fornaciari@med.unipi.it (G.F.); v.giuffra@med.unipi.it (V.G.); 5 Center for Anthropological, Paleopathological and Historical Studies of the Sardinian and Mediterranean Populations, Department of Biomedical Sciences, University of Sassari, 07100 Sassari, Italy; 6 Laboratory of Molecular Archaeo-Anthropology/Ancient DNA, School of Biosciences and Veterinary Medicine, University of Camerino, 62032 Camerino, Italy; stefania.luciani@unicam.it (S.L.); isolina.marota@unicam.it (I.M.); 7 Environmental Microbiology Laboratory, Department of Biology, University of Puerto Rico, San Juan 00932, Puerto Rico; gary.toranzos@upr.edu

**Keywords:** carbohydrate-active enzymes, gut microbiome, mummies, resistome

## Abstract

Little is still known about the microbiome resulting from the process of mummification of the human gut. In the present study, the gut microbiota, genes associated with metabolism, and putative resistome of Inca and Italian nobility mummies were characterized by using high-throughput sequencing. The Italian nobility mummies exhibited a higher bacterial diversity as compared to the Inca mummies when using 16S ribosomal (rRNA) gene amplicon sequencing, but both groups showed bacterial and fungal taxa when using shotgun metagenomic sequencing that may resemble both the thanatomicrobiome and extant human gut microbiomes. Identification of sequences associated with plants, animals, and carbohydrate-active enzymes (CAZymes) may provide further insights into the dietary habits of Inca and Italian nobility mummies. Putative antibiotic-resistance genes in the Inca and Italian nobility mummies support a human gut resistome prior to the antibiotic therapy era. The higher proportion of putative antibiotic-resistance genes in the Inca compared to Italian nobility mummies may support the hypotheses that a greater exposure to the environment may result in a greater acquisition of antibiotic-resistance genes. The present study adds knowledge of the microbiome resulting from the process of mummification of the human gut, insights of ancient dietary habits, and the preserved putative human gut resistome prior the antibiotic therapy era.

## 1. Introduction

Ancient human microbiomes are preserved due to specific taphonomic conditions. While dental calculus, bones, and coprolites represent valuable sources of ancient microbial DNA [1,2,3,4,5,6,7,8], mummified human gut remains represent a unique opportunity to further explore the microbiome. The process of natural mummification is highly unique, generally resulting from specific conditions including, but not limited to, the combination of low or elevated temperatures, low oxygen levels and dry weather conditions [9]. On the other hand, the process of artificial mummification, or embalmment, is an invasive method of preservation of the body that would include evisceration, and filling the body with substances. In both types of mummification, decomposition, and putrefaction is minimized or halted [10]. 

Ancient cultures in the Americas and Europe practiced the process of mummification by intentionally exposing the bodies to the necessary environmental conditions. For instance, in pre-Inca and Inca societies, bodies would be generally mummified in chamber tombs, and were not buried [10,11]; thus, the body would not go through the process of decomposition aided by soil microorganisms. Comparably, Italian nobility bodies would be preserved by “dripping” or removing fluids from the bodies prior their placement in empty spaces that possessed the necessary conditions for the process of natural mummification to occur [12]. The process of “dripping” the body is essential to halt the process of putrefaction. Preservation of Italian nobility bodies may have been favored by the particular microclimatic conditions of certain geographical regions in Italy, as well as by the disposition of the coffins near the windows at about 5 m of height [13]. 

The recoverability of nucleic acids from diverse bacteria, fungi, microbial eukaryotes (e.g., *Trypanosoma cruzi* and *Leishmania donovani*), and viruses (e.g., bacteriophages and Human Papillomavirus (HPVs)) in pre-Inca (10–11th century) and Inca (14–15th century) mummies has previously been reported [14,15,16,17,18]. In the present study, the recoverable gut microbiome, as well as metabolic genes and putative resistome profiles of previously characterized pre-Inca/Inca mummies, as well as four natural and one artificial Italian nobility mummies from the Renaissance of Naples period (15–16th centuries) were characterized. These mummies are unique in that they exhibited diverse pathologies, lifestyles, dietary habits, exposure to the environment and socioeconomic status. Pre-Inca and Inca societies were in a constant and direct contact with the environment, and were accomplished travelers, builders and farmers. Pre-Inca and Inca diets were mainly plant-based and included quinoa, maize, and potatoes [19]. Meat was rarely consumed and may have included guinea pigs, as well as llama and alpaca herds, which were consumed as charque, a form of jerky from South America [20,21]. The most common drink was chicha, which was made from fermented maize, quinoa or other grains [21]. Diets of Italian nobility from the 15–18th centuries, on the other hand, may have been primarily animal-based. Historical and isotopic records have shown that meat constituted a central part of the nobles’ diet, and fish was consumed on penitential occasions [22]. Although less frequently, eggs and cheese were also consumed [23]. Fruits and vegetables were not commonly consumed or were absent from the Italian nobility’s diet [22,24] (Supplementary Table S1). Results could represent an opportunity to infer the effect of the process of natural and artificial mummification to the gut microbiota structure, as well as to the sequences that are associated with metabolism and the putative resistome, which were not investigated in detail in previous studies. Similar to recent studies inferring dietary habits and behavior of Neanderthals from the dental calculi microbiome, data from mummified gut remains could be also potentially used to infer ancient dietary habits [25]. 

## 2. Materials and Methods

### 2.1. Description of the Pre-Inca and Inca Mummies

All of the mummy samples were acquired with all of the necessary permissions from the Division of Paleopathology at the University of Pisa. The pre-Inca/Inca mummies are at the Museum of Anthropology of the University of Florence (FI3 and FI9) and in the Museum of Pathology of the University of Pisa (FI12). The Inca mummies were exhumed and brought to Italy in the second half of the 19th century. The Italian mummies were placed in wooden sarcophagi in the Sacristy of the Basilica of St. Domenico Maggiore in Naples at the end of explorations in 1992. Autopsy was performed to all mummies by paleopathologists wearing sterile surgical coats, sterile latex gloves, sterile masks, headdresses, and overshoes. Colon or abdominal viscera samples were collected directly from tissue. 

All pre-Inca/Inca mummies were naturally mummified. Mummy FI9 was a female of estimated age 18–23 years from Cuzco, Peru, with a ^14^C dating to 980–1170 A.D. (pre-Inca period) [8]. Sequences from the descending colon were included in the present study. In addition, a paleopathological study of this mummy demonstrated several different phenotypic abnormalities related to Chagas’ disease, caused by *Trypanosoma cruzi*, as a possible cause of death [26]. The characteristics pointing to the disease in its chronic phase included cardiomegaly, mega-esophagus, gastric ectasia, and megacolon with enormous amounts of feces, fibrosis of the myocardium, esophagus, and colon. Mummy FI3 was a Peruvian adult male dating to the 14–15th century, from Cuzco, Peru, and was approximately 25 years. DNA was extracted from abdominal viscera, but a complete autopsy of this mummy was not possible; however, the mummy showed a good preservation of the skin with the adnexa, and a massive presence of fungi and ectoparasites (Marazzini et al., 2015). Mummy FI12, with a ^14^C dating to 1410–1530 A.D. was an adult female of estimated age of 20–25 years, and autopsy showed that she was affected by bronchopneumonia. Both lungs, as well as the upper right bronchus, were easily identified and were sampled during the autopsy. The right lung showed numerous alveoli, in large fibrous areas, with well recognizable vessels and bronchi and widespread areas of pulmonary anthracosis. An important element is the massive presence of alveolar exudate. From the fetal position from burial in a basket, it is evident that it may be from the Inca culture (15–16th centuries). DNA was extracted from the transverse colon [16]. It should be highlighted that the characterized pre-Columbian Andean mummies included in the present study were not buried, as they were preserved in funerary crypts; thus, they did not go through the process of decomposition aided by soil microorganisms (Supplementary Table S1).

### 2.2. Description of the Renaissance Mummies from Naples 

The majority of the Italian mummies (NASD3, 14, 27, 29) are natural (not embalmed) and were deposed in small rooms (“colatoi”), provided with a horizontal grill made of wood or pottery tubules, on which the corpses were placed to slowly lose their liquids through the skin. Ventilation was made possible by simple air flow systems, guarantying the drying of the tissues. Italian mummy NASD22 (King Ferrante I) was the only artificial mummy, and it was eviscerated and filled with marine sponges and vegetal resins, with exception of the rectum. Mummy NASD3, identified as Pietro of Aragona, III Duke of Montalto was 12 years old (ca. 1539–1552), and showed thoracic and abdominal organs collapsed to the posterior thoracic and abdominal walls, with still visible mediastinum and diaphragm, and lungs reduced with well identifiable bronchial tree. The heart was significantly reduced to some millimeters thick, but showed typical morphology, and appeared as attached to the thoracic column. The stomach was of typical morphology, but reduced to a leaf of some millimeters. Small and large bowel were totally dry and collapsed to the posterior abdominal wall, but were still well recognizable. It was possible to dissect and isolate the left kidney, which was totally flat but still with the ureter [27]. DNA was extracted from transverse colon. Mummy NASD14, identified as Ferdinando Orsini, V Duke of Gravina, dates to the 16th century and was 50–55 years old. DNA was extracted from the small bowel. The mummy showed a large, destructive basal cell carcinoma (*ulcus rodens*) of the right orbit and nose [28,29,30,31,32]. Autopsy showed completely dehydrated thoracic organs collapsed to the posterior thoracic wall. Mediastinum, diaphragm, trachea, and primary bronchi were well preserved, while the heart and the lungs appeared like thin sheets. The liver had reduced size (10 cm diameter), and fibrous consistency but typical form and falciform ligament still identifiable. The stomach was small but well recognizable. The large and small intestines, reduced to thin sheets, were attached to the posterior abdominal wall in the correct anatomical position, with the cecum being well identifiable. Bladder and rectum were still in situ. The aortic arch, and thoracic and abdominal aorta were easily recognizable [28,31]. Artificial mummy NASD22, identified as the king of Naples Ferrante I of Aragon, dates to the 15th century and was 70 years old. DNA was extracted from rectum with feces. The mummy revealed a hollow fibrous structure of the small pelvis, identified as rectum at autopsy and at X-ray. Histology revealed epithelial tumor cells, disposed in cords, solid nests and glands, disseminated in a fibrous stroma containing striated muscular fibers. Cells were crowded and tall, with abundant cytoplasm and pseudo-stratified pleomorphic hyperchromatic nuclei. The mucus was scarce and limited to pseudo-glandular formations, as shown by the specific staining. These results pointed out a moderately differentiated mucinous adenocarcinoma infiltrating the muscular-fibrous layers of the small pelvis. The histological, histochemical, and immunohistochemical results clearly indicate a mucinous adenocarcinoma of the digestive tract, most probably of the colon rectum. DNA hybridization probe and sequencing demonstrated a *K-ras* exons 1–2 punctiform mutation, the most frequent mutation of the *K-ras* gene in sporadic colorectal cancer, as in the case of the king, is associated with high consumption of red meat, which is known to induce a significant increase in fecal carcinogenic N-nitroso compound (NOC) levels [32,33,34]. Mummy NASD27, identified as Luigi Carafa, II Prince of Stigliano, dates to the 16th century and was 65 years old. DNA was extracted from intestine. The mummy showed well evident mediastinum and diaphragm, with well recognizable trachea still in situ, but the lungs and heart were not evident. Stomach was of typical morphology, but reduced to a leaf. Intestinal loops were identifiable, but damaged. The liver was partially preserved, but was reduced to a small, hard mass. After complete dissection of thoracic and abdominal organs, the thoracic aorta was well visible. Mummy NASD29 was an unknown adult male dating to the 16th century and was 20–25 years. DNA was extracted from colon. The mummy revealed well preserved thoracic and abdominal organs, dried and partially collapsed on the posterior wall. The pericardial sac contained the heart, which was wrinkled (7 × 4 cm) and flattened; the mediastinum and the diaphragm were clearly identified, as well as the bronchial tree and lungs. The liver was reduced in size (18 × 9 cm), but was of typical shape, with the falciform ligament and the hepatic lobes being easily recognizable, and a granular surface with a large number of single or clumped 0.5–1.5 cm nodules. The kidneys, rightly positioned, were typically shaped (7 × 3 cm) and appeared flattened up to 0.5 cm. Liver histology evidenced coarse bands of fibrous tissue dissecting and surrounding areas of homogeneous, brown and granular material, representing residual hepatic parenchyma, and resulting in a sort of pseudo-lobular organization. Other nodular formations, with multi-layered fibrous connective scars, surrounding regions of wholly degenerated parenchyma, were evidenced. The macroscopic and microscopic pictures were consistent with the diagnosis of micro-macronodular cirrhosis [35]. The histological study of the colon revealed an extraordinarily well-preserved colon mucosa with an evident villous adenoma and strong immune-positivity for keratins and p53.1. In some points, clear invasion of the polyp stalk or of the submucosa was evident: the histological picture is that of a well differentiated adenocarcinoma at stage T1 [31,32]. Importantly, after dripping of the bodies, the mummies were preserved in empty spaces, as described above (Supplementary Table S1).

### 2.3. DNA Extraction and Avoidance of Contamination

Tissue samples to be used for DNA extraction were prepared from whole internal organs and were removed during the autopsy of the mummies and which had been stored aseptically. The mummified specimens were immediately kept and sealed in sterile containers, reducing the opportunities for subsequent contamination. The samples were stored aseptically in hermetic plastic containers, in a dry environment with silica gel at 18–20 °C. The outermost portions of the specimens were discarded to eliminate the risk of contamination and one replicate per sample type was obtained for further analyses. We employed the standard precautions for ancient DNA work including the use of sterile gloves, pretreatment of mortars, pestles, and homogenizers with HCl (2N), use of UV-irradiated safety cabinets, dedicated gel trays, tanks, and reagents. DNA extractions were conducted in the laboratory of Molecular Archaeo-Anthropology/ancient DNA in the University of Camerino (Italy), under strict rules to prevent DNA contamination. The ancient DNA laboratory was constructed exclusively for ancient DNA and no molecular analyses have ever been performed on modern DNA. The laboratory comprises an antechamber, in which the operator wears a full body sterile suit, gloves, a face screen, and an extraction room equipped with UV lights, and a positive-pressure air-filtering system providing a High Efficiency Particulate System (HEPA) filtration system with a complete change of air every 10 min.

DNA was extracted from approximately 0.2 g of the tissue samples using the phenol-chloroform method, as described previously [14]. Non-template or blank controls were added and processed following the same protocol in order to detect any potential contamination from reagents or the extraction process. The quality of the DNA was checked in agarose gels, showing no high-molecular weight bands, indicating DNA fragmentation (dozens to 200–300 bp), consistent with the integrity of authentic ancient DNA [14]. 

### 2.4. 16S ribosomal RNA Gene High-Throughput Sequencing

DNA amplification of the 16S ribosomal (rRNA) gene was performed at Molecular Research Laboratory (MRDNA) (www.mrdnalab.com; Shallowater, TX, USA). All of the DNA samples were handled in exclusive areas for Polymerase Chain Reaction (PCR) amplification, which are sterilized before and after every use using DNAaway and UV-radiation to eliminate cross-contamination with modern samples. Template manipulations are handled in separate hoods that are sterilized before and after every manipulation using DNAaway and UV-radiation. Negative PCR controls were included in all of the amplification reactions, showing no contamination. The 16S rRNA gene V4 variable region was amplified using the PCR primers 515f (GTGCCAGCMGCCGCGGTAA)/806r (GGACTACHVGGGTWTCTAAT). PCR amplifications were conducted using a single step 30 cycle PCR using the HotStarTaq Plus Master Mix Kit (Qiagen, Valencia, CA, USA) under the following conditions: 94 °C for 3 min, followed by 30 cycles of 94 °C for 30 s, 53 °C for 40 s, and 72 °C for 1 min, after which a final elongation step at 72 °C for 5 min was performed. The V4 regions was chosen as it has been more characterized when compared to other regions, and provides more reliable results as compared to other hypervariable regions [36]. The size of the V4 region is within the appropriate length for ancient DNA analyses. After amplification, PCR products were checked in a 2% agarose gel to determine the success of amplification and the relative intensity of the bands. All amplicon products from each sample were mixed in equal concentrations and purified using Agencourt AMPure beads (Agencourt Bioscience Corporation, Beverly, MA, USA). Mummies FI3, FI12, NASD3, NASD14, NASD22, NASD27, and NASD29 16S rRNA gene libraries were created using the Ion Plus Fragment Library Kit following the manufacturer’s instructions. Libraries were then sequenced using 314 chips on an Ion Torrent Personal Genome Machine (PGM; Life Technologies, Grand Island, NY, USA). Mummy FI9 DNA was previously prepared in a DNA library following Illumina MiSeq DNA library preparation protocol using the MiSeq reagent kit V2 (Illumina Inc., San Diego, CA, USA) (2 × 150 bp) for paired-end reads on a MiSeq following the manufacturer’s guidelines [16]. 

### 2.5. 16S ribosomal RNA Gene Sequence Analyses 

Paired-end fastq files were joined using the QIIME pipeline using join_paired_ends.py. Barcodes were then removed using split_libraries.py with default filtering parameters (http://qiime.org/scripts/split_libraries.html). 16S rRNA gene sequences were sorted based on sample ID using the Quantitative Insights Into Microbial Ecology (QIIME) script extract_seqs_by_sample_ id.py. Operational taxonomic units (OTUs) were selected using pick_open_reference_otus.py workflow [37]. 16S rRNA taxonomy up to the genus level was defined by ≥97% similarity to reference sequences in the Greengenes database. Bayesian microbial source tracking was performed to identify possible sources of contamination, particularly those matching human skin microbiomes in case the samples were not properly handled, and/or soil microbiomes given that these samples have been in contact with the environment to some extent. Notably, pre-Inca and Inca bodies were mummified in funerary crypts, and were not buried; thus, the body would not go through the process of decomposition aided by soil microorganisms. Comparably, Italian nobility bodies would be preserved by “dripping” or removing fluids from the bodies prior their placement in empty spaces that possessed the necessary conditions for the process of natural mummification to occur. Sources for the analysis included human (45 gut, 45 oral and 45 skin) and non-human (45 soil) microbiomes, for a total of 180 microbiomes downloaded from the SourceTracker tutorial (http://qiime.org/tutorials/source_tracking.html). Alpha and beta diversity, alpha rarefaction curves, and taxonomy assignments were determined using the core_diversity.py workflow. Data were rarefied to 24,600 sequences to minimize the effect of disparate sequence number on the results. Alpha diversity metrics of the bacterial communities, including Phylogenetic Diversity (PD) whole tree, chao1, and observed species were computed from the average of ten iterations from the alpha collated results. Alpha diversity between the pre-Inca/Inca and Italian nobility mummies was as compared using the compare_alpha_diversity.py script with default parameters, including a nonparametric two sample *t*-test, and using Bonferroni correction. Procrustes plots were constructed in QIIME using the transform_coordinate_matrices.py script, and the principal coordinate files of the unweighted and weighted unifrac metrics, followed by the make_emperor.py scripts, with default parameters. Unweighted and weighted unifrac metric values between the pre-Inca/Inca and Italian nobility mummies were compared using the compare_categories.py script. An analysis of similarity (ANOSIM) was used to compare the pre-Inca/Inca and Italian nobility mummies. The shared_phylotype.py and group_significance.py scripts were used to determine the quantity and identity of the OTUs shared between the mummies, respectively. OTU frequencies were compared between the pre-Inca/Inca and Italian nobility mummies using the group_significance.py script and Kruskal-Wallis (default) comparisons because no assumptions are made when applying this test (http://qiime.org/scripts/group_significance.html). Only Bonferroni-corrected *p*-values ≤ 0.05 were considered as significant. Taxonomic analyses up to the genus level were performed with the non-template control. Briefly, OTUs were picked using the pick_open_reference_otus.py workflow, followed by taxonomic classification using the summarize_taxa_through_plots.py script. 

### 2.6. Shotgun Metagenome Sequence Processing

DNA preparation for metagenome sequencing was also performed at MRDNA under strict procedures to eliminate cross-contamination with modern DNA including working in areas that are sterilized before and after every use using DNAaway, as well as UV-radiation to eliminate cross-contamination with modern samples. DNA library for metagenome analyses was prepared following the Illumina MiSeq DNA library preparation protocol using the MiSeq reagent kit v2 (2 × 100 bp) for paired-end reads on a MiSeq following the manufacturer’s guidelines. Mummy FI9 DNA was previously prepared for shotgun metagenomic sequencing, as previously described (Santiago-Rodriguez, Fornaciari et al., 2015). Barcodes were trimmed using a proprietary analysis pipeline from MRDNA. Fastq files were imported into CLC Genomics Workbench as Paired-reads (where it is assumed that the first reads of the pairs are in one file and the second reads of the pairs to be in another), with a minimum and maximum distance of 50 and 250, respectively, with forward orientation and removing failed reads. Paired reads were then used for all the metagenome analyses. Human DNA sequences were removed using a proprietary software of MRDNA. We trimmed each read according to Phred- or Q-scores of 0.5, removed any low complexity reads with ≥8 consecutive homopolymers, and removed any reads with substantial length variation (<50 nucleotides) or ambiguous characters (N’s) prior to further analysis using CLC Genomics Workbench. CLC Genomics Workbench recommended trimming quality score is 0.05, where the lower the value the more stringent the analysis is (http://resources.qiagenbioinformatics.com/manuals/clcgenomicsworkbench/current/index.php?manual=Quality_trimming.html). Quality scores in CLC Genomics Workbench are in Phred scale and are defined as: Q = −10log_10_ (P), where P is the base-calling error probability. Therefore, low probability points to high quality bases. Reads were assembled using the De novo Assembly option in CLC Genomics Workbench with the following parameters: Mapping mode = Map reads back to contigs (slow); Update contigs = Yes; Automatic bubble size = Yes; Minimum contig length = 100; Perform scaffolding = Yes; Auto-detect paired distances = Yes; Mismatch cost = 2; Insertion cost = 3; Deletion cost = 3 using an 80% identity over a 50% of the read length. MapDamage analyses were performed with the contigs as described previously. Briefly, reads were retrieved from CLC Genomics Workbench as a SAM file, and were processed using mapDamage for further ancient DNA authentication, as described previously https://ginolhac.github.io/mapDamage/ [38].

### 2.7. Taxonomic Assignments of Shotgun Metagenomic Data

Metagenome binning was performed with the contigs in order to assign sequences to a taxonomic group at the order level. Metagenomes were analyzed for taxonomy assignments by sequence composition-based classification using the Classifier for Metagenomic Sequences (ClaMS) (http://clams.jgi-psf.org/), which only considers similarity to assign sequences to a user-specified taxonomic level, and which also utilizes the Integrated Microbial Genomes (IMG) database [39,40]. Metagenome binning was conducted using the de Bruijn chain signature (DBC) with a k-mer length of 4 and a distance cut-off value setting of 0.07. Binning results were exported as text and the results were analyzed using Microsoft Excel to sort and count the number of repetitions for a particular taxon. A relative abundance of taxa were calculated for each metagenome and plotted [41]. Linear Discriminatory Analysis (LDA) effect size (LEfSe) pipeline, available at http://huttenhower.sph.harvard.edu/galaxy/ was used to estimate the effect size of each differentially abundant taxa. Alpha values of 0.05 were used and a threshold of 2.0 was chosen for logarithmic LDA scores.

### 2.8. Sequences Associated with Diet, Metabolic Profiles and Carbohydrate-Active Enzymes

To determine the proportion of sequences associated with plants and animals, contigs were uploaded to DiaGrid (Purdue Research Foundation, West Lafayette, IN 47906, USA) BLASTer tool (https://diagrid.org/tags/blasterupload), BlastX against the NCBI database and annotated with a minimum identity and e-value cutoff of 80% and 1.0 × 10^−15^, respectively. The 1.0 × 10^−15^ value is sufficiently high to filter out weaker homologies, but not high enough to filter out potentially interesting hits. The resulting matrix was then filtered based on taxonomy (L3, L4, and L5) and were examined for each taxonomic group (at genus, species, and common name level). Interesting hits were evaluated based on query length of reads (≥50 aa) and percent homology of 95–100%. The taxonomic identification of the “diet” components were made on the preponderance of high quality hits (≥10) for metabolic and other functional genes. These must also have had high read length (≥50 aa) and % homology (≥95%) for the same taxon. Results were exported as text and the results were sorted using Microsoft Excel to count and plot the number of repetitions for a particular plant or animal scientific classification. Plant and animal categories were manually classified into common names. Metabolic categories were determined based on homologies to gene categories in the KEGG database. For this, data were uploaded and annotated using the Metagenomic Rapid Annotations using Subsystems Technology (MG-RAST) pipeline with a minimum e-value cutoff of 1.0 × 10^−5^ and a minimum identity cutoff of 80% [41]. Kegg Orthology (KO) counts were normalized using total sum normalization. Analysis of variance (ANOVA) single factor was performed in Excel with an alpha value of 0.05 to compare the CAZyme categories. *p*-values were adjusted for multiple comparisons using the Bonferroni correction. A false discovery rate of <0.05 was considered as statistically significant. A heatmap of the Euclidean distances of normalized KO counts associated with carbohydrate, amino acid, and lipid metabolism was constructed using custom scripts and the function heatmap.2 from the gplots package in the R statistical software v 3.3.2. When using the default methods for the heatmap.2() function in R, the distance measure is calculated using the dist () function, whose default is Euclidean distance. Clustering is a graphical representation of the similarities and differences of the Euclidean distances. Pearson correlation coefficients were then calculated using JMPPro to identify potential correlations between KOs and taxonomy at the order level. The correlation coefficient matrix was then exported and a correlation heatmap was constructed using custom scripts and the lattice package in the R statistical software v 3.3.2. 

To determine the proportions of CAZyme genes, contigs were uploaded to http://www.bioenergycenter.org/besc/index.cfm, and were annotated using a minimum identity and e-value cutoff of 80% and 1.0 × 10^−5^. Results were exported as text and the results were sorted using Microsoft Excel to count the number of repetitions for a particular taxon and CAZyme family. Taxon categories were manually classified at the phylum level. Data were also compared to modern Amazonians and Italians. Raw sequence files from the gut metagenome of two modern adult female Amazonians of 35 and 29 years, respectively, (MG-RAST IDs 4461123.3 and 4461130.3) were downloaded from MG-RAST. Contig files from the gut metagenomes of two modern Italians, a male and a female of 38 years and 34 years old, respectively (MG-RAST IDs 4560437.3 and 4560441.3) were downloaded from MG-RAST. CAZyme counts were normalized using total sum normalization. Heatmaps of the Euclidean distances of the normalized CAZyme gene family counts were constructed as described above. ANOVA single factor was performed in Excel with an α value of 0.05 to compare CAZyme categories. *p*-values were adjusted for multiple comparisons using the Bonferroni correction. A false discovery rate of <0.05 was considered as statistically significant.

### 2.9. Putative Resistome Characterization 

For putative resistome characterization, contigs were screened against the Comprehensive Antibiotic Resistance database (CARD) https://card.mcmaster.ca/ using BLASTx with an e-value cutoff of 1.0 × 10^−5^. The abundance of each target gene was retrieved, parsed using IonAssist (http://www.thepridelaboratory.org/software.html), and normalized by the total number of contigs in the correspondent sample. Bar plots of the normalized gene counts were constructed. Analysis of variance (ANOVA) single factor was performed in Excel with an α value of 0.05 to compared categories. *p*-values were adjusted for multiple comparisons using the Bonferroni correction. A false discovery rate of <0.05 was considered as statistically significant.

## 3. Results

### 3.1. Characterization of the Mummies

Gut tissues from the mummies described in Supplementary Table S1 and Materials and Methods Section, were sampled using all necessary precautions for ancient DNA analyses (see Materials and Methods). The 16S rRNA gene hypervariable region 4 (V4) was sequenced, resulting in 24,731 (mummy FI9); 78,903 (mummy FI3); 56,971 (mummy FI12); 32,086 (mummy NASD3); 44,255 (mummy NASD14); 60,266 (mummy NASD22); 24,633 (mummy NASD27); and, 101,517 (mummy NASD29) sequences after quality filtering http://qiime.org/tutorials/usearch_quality_filter.html.

### 3.2. Characterization of the 16S ribosomal RNA Gene by High Throughput Sequencing

An average of 302 ± 52 and 710 ± 129 OTUs at ≥97% identity was observed in the pre-Inca/Inca and Italian nobility mummies, respectively, and a number of these were shared between the groups (Supplementary Table S2). Prior any further analyses, Bayesian Source Tracker analyses were performed to identify potential sources of environmental contaminant DNA, including that originating from soils [42]. Mummy FI3 had the highest number of sequences (>85%) matching extant gut microbiomes; yet, the majority of the sequences (>95%) in the remaining mummies did not resemble any of the microbiomes that were included in the analyses (i.e., stool, oral, skin, and soil) (Supplementary Figure S1). Data were then rarefied to 24,600 sequences to minimize the effect of disparate sequence number on the results. Chao1 (Supplementary Figure S2A) observed OTU counts (Supplementary Figure S2B) and Phylogenetic Diversity (PD) whole tree (Supplementary Figure S2C) rarefaction curves showed that data reached or were close to reaching a plateau when rarefying data to 24,600 sequences. Three different metrics were used to calculate alpha diversity including Chao1 for microbial richness (Supplementary Figure S2D), observed OTUs counts (Supplementary Figure S2E) and PD whole tree (Supplementary Figure S2F). All of the measures indicate a higher alpha diversity within the Italian nobility mummies compared to the pre-Inca/Inca mummies (nonparametric two sample *t*-test; Bonferroni adjusted *p* < 0.01). 

**Figure 1:**
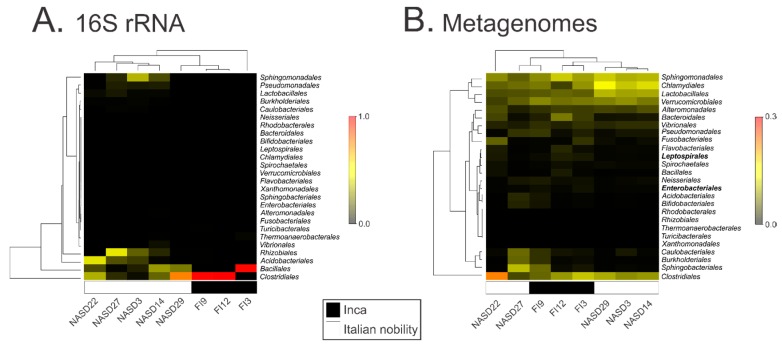
Characterization of the gut microbiome of the pre-Inca/Inca and Italian nobility mummies. (**A**) Heatmap of the Euclidean distances of the relative abundances of the 16S ribosomal RNA (rRNA) gene and (**B**) shotgun metagenomics sequencing data. 16S rRNA gene data were annotated using Quantitative Insights Into Microbial Ecology (QIIME) with a minimum identity cutoff of 97%. Metagenomes were annotated using CLaMs http://clams.jgi-psf.org/. Classification was performed at the order level with a minimum identity cutoff of 80% and e-value of 10 × 10^−5^.

The gut microbiota of the pre-Inca/Inca and Italian nobility mummies are significantly different (ANOSIM; *p* = 0.014), with data clustering according to culture, as shown with both the heatmap (Figure 1A), and the Procrustes plot (Supplementary Figure S3). Taxonomy results were presented at the order level given that the majority of the sequences at the phylum level in the Pre-Inca/Inca (>98.0%) and Italian nobility (>52.5%) mummies, with the exception of NASD3 (14.3%) and NASD27 (18.5%), corresponded to the Firmicutes. Taxonomy at the order level still enabled a degree of identification, as a number of OTUs could not be assigned at the genus level (Supplementary Table S3). The remaining sequences for mummies NASD3 (68.6%) and NASD27 (64.7%) corresponded to Proteobacteria. The pre-Inca/Inca and Italian nobility mummies exhibited differences in microbiota relative abundance at the order level. While mummies FI9 (97.6%) and FI12 (99.6%) had a higher representation of *Clostridiales*, mummy FI3 was characterized by having a higher representation of *Bacillales* (96.8%). Interestingly, the *Bacillales* and *Clostridiales* were not the most represented taxa in most of the Italian nobility mummies. Unlike the pre-Inca/Inca mummies, there was no clear pattern on the bacterial relative abundances in the Italian nobility mummies. For example, while *Clostridiales* comprised the majority of the bacterial communities in mummy NASD22 (34.0%) and NASD29 (73.9%), *Sphingomonadales*, *Bacillales*, and *Rhizobiales* comprised the majority of the bacterial communities in mummies NASD3 (35.1%), NASD14 (29.7%), and NASD27 (44.9%), respectively, which are also known to be members of modern human gut microbiomes [43,44]. Other bacterial taxa were also identified in smaller relative abundances, depending on the mummy (Figure 1A and Supplementary Table S3). LEfSe did not identify any OTUs being significantly different between the mummies, certain bacterial groups including the *Alteromonadales*, *Bifidobacteriales*, *Flavobacteriales*, *Rhodobacteriales*, *Sphingobacteriales*, and *Xanthomonadales* were exclusively present in the Italian nobility mummies in small relative abundances (<1.0%). Specific bacterial genera including, but not limited to, *Corynebacterium* spp. (OTU 2590) (Kruskal-Wallis test; Bonferroni-adjusted *p* = 0.018) and *Staphylococcus* spp. (OTU 1113297) (Kruskal-Wallis test; Bonferroni-adjusted *p* = 0.018) appeared to be depleted in the Inca mummies, as shown with the group significance analysis (Supplementary Dataset S1). The Italian nobility mummies also appeared to be depleted of specific bacterial genera including, but not limited to *Turicibacter* spp. (OTU 2394) (Kruskal-Wallis test; Bonferroni-adjusted *p* = 0.033), and various OTUs classified as *Clostridium* spp. (Supplementary Dataset S1). Thirty-nine OTUs were detected in the non-template or blank control, with the *Pseudomonadales* and *Burkholderiales* being the most abundant orders, representing 20.4 and 15.4% of the OTUs, respectively (Supplementary Dataset S2). 

### 3.3. Characterization of the Gut Microbial Communities Using Shotgun Metagenomics

The gut microbial communities in the pre-Inca/Inca and Italian nobility mummies were also characterized using shotgun metagenomics, generating 146,081,692 reads for mummy FI9; 16,805,260 reads for mummy FI3; 16,537,474 reads for mummy FI12; 16,570,556 reads for mummy NASD3; 17,687,976 reads for mummy NASD14; 18,959,490 reads for mummy NASD22; 16,847,766 reads for mummy NASD27; and, 14,265,734 reads for mummy NASD29. Results did not show nucleotide misincorporation patterns characteristic of ancient DNA when performing the mapDamage analysis (Supplementary Figure S4). Representative results are shown in Supplementary Figure S4, although similar outcomes were obtained for all the mummies, where substitutions from C to T, and G to A were not restricted to the 5′ and 3′ ends, respectively. All of the further analyses were performed using the assembled reads (see Materials and Methods) (Supplementary Table S4). Shotgun metagenome analyses revealed other bacteria, as well as archaeal and fungal communities in the pre-Inca/Inca and Italian nobility mummified gut remains. *Clostridiales* exhibited the highest relative abundance ranging from 4.9 to 33.8%, depending on the mummy. Other microbial taxa present in high relative abundances included the *Sphingobacteriales*, *Chlamydiales*, *Verrucomicrobiales*, and *Lactobacillales*. LEfSe results showed that the *Leptospirales* and *Enterobacteriales* were unique features of the guts of the pre-Inca/Inca mummies (highlighted in bold) (Figure 1B). 

### 3.4. Sequences Associated with Dietary Habits and Metabolism 

**Figure 2:**
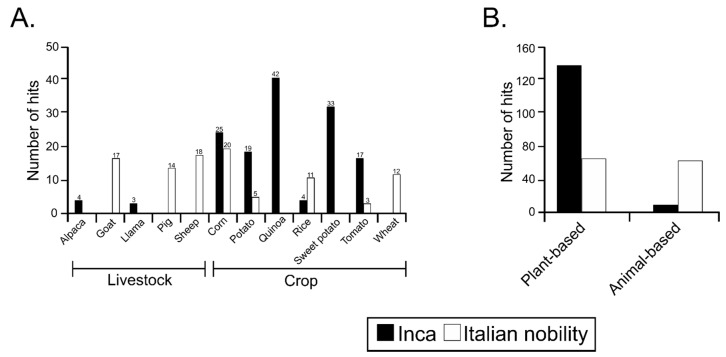
Relative proportions of plant and animal sequences identified in the guts of pre-Inca/Inca and Italian nobility mummies. (**A**) Number of sequence hits associated with livestock including alpaca, goat, llama, pig and sheep, and crops including corn, potato, quinoa, rice, sweet potato, tomato and wheat; (**B**) Sequence hits were then classified as plant- or animal-based diets. Contigs were uploaded to DiaGrid BLASTer tool (https://diagrid.org/tags/blasterupload) and annotated using a minimum identity of 80% and e-value of 10 × 10^−5^. Plant and animal sequences were then manually curated to their common names.

Shotgun metagenomics data were analyzed to explore the sequences that were associated with plants and animals as these may be useful to infer dietary habits of ancient cultures. Common names were used, instead of scientific names, mainly because of the attempt to describe the broad dietary components rather than species. Many of the modern “species” were probably not of the same genetic line 500–1000 years ago; thus, using specific epithets would be misleading and would potentially lose valuable dietary information. Using the potato and quinoa as examples, the Incas developed a multitude of varieties, all of which would have different genetic profiles. Sequences associated with alpaca and llama were found to be exclusively present in the pre-Inca/Inca culture, whereas sequences associated with goat, pig, and sheep were primarily present in the Italian nobility mummies. Sequences associated with crops such as corn, potato and tomato were present in both the pre-Inca/Inca and Italian nobility (with the exception of Ferrante I of Aragon, mummy NASD22) mummified guts, with a higher number of hits present in the pre-Inca/Inca mummies. Sequences associated with rice were present in both cultures, but were more represented in the Italian nobility mummies. The small number of rice-associated sequences in the pre-Inca/Inca culture may possibly belong to the genus *Oryza* (*grandiglumis* or *latifolia*), present in Peru, and occasionally used as an alternative food source [45,46]. Quinoa- and sweet potato-associated sequences were exclusively present in the pre-Inca/Inca culture, whereas sequences associated with wheat were exclusively present in the Italian nobility mummies (Figure 2A). When sequences were classified as a plant- or animal-based diet, the pre-Inca/Inca culture was characterized by having a higher number of sequences associated with a plant-based diet, whereas the Italian nobility was characterized by having a higher number of sequences associated with an animal-based diet, when compared to the pre-Inca/Inca, consistent with the archeological evidence (Figure 2B). 

**Figure 3:**
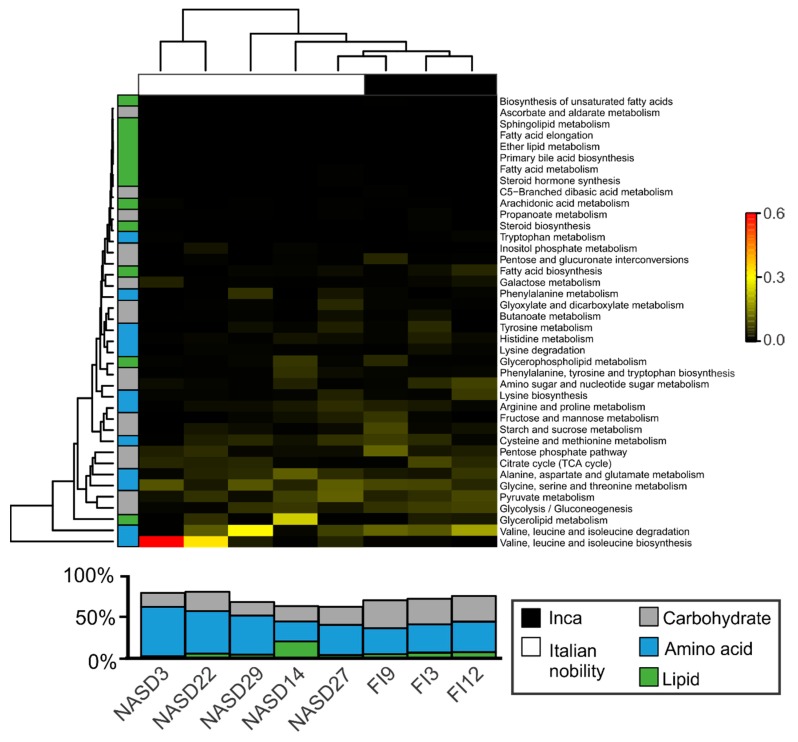
Characterization of the gut functional profiles associated with carbohydrate, amino acid and lipid metabolism in the pre-Inca/Inca and Italian nobility mummies. The top panel shows a heatmap of the Euclidean distances of the Kegg Orthology (KO) counts associated with carbohydrate, amino acid and lipid metabolism. The bottom panel shows the relative abundance of carbohydrate, amino acid and lipid metabolism. Contigs were uploaded to Metagenomic Rapid Annotations using Subsystems Technology (MG-RAST) and annotated using the KEGG database with a minimum identity of 80% and an e-value of 10 × 10^−5^.

Pre-Inca/Inca and Italian nobility mummies metagenomes showed a comparable functional configuration dominated by genes associated with metabolism including sulfur, protein, RNA, potassium, and nitrogen metabolism, although differences between the cultures did not meet statistical significance (Supplementary Figure S5). A total of 2723 KO was further analyzed, of which 78 and 392 were unique to the Inca and Italian nobility mummies, respectively, and 1550 were shared between both cultures. Given that carbohydrate, amino acid and lipid metabolism comprised >50% of the metabolic categories of the pre-Inca/Inca and Italian nobility mummies, associated KOs were further explored. Results showed that that there was an overall separation of the KO profiles based on culture. While Glycolysis/Gluconeogenesis (K00010) was significantly more represented in the pre-Inca/Inca (7.2% ± 0.6%) as compared to the Italian nobility (3.3% ± 1.0%) mummies (ANOVA; *p* = 0.043), the remaining categories were virtually similar (Figure 3). 

In the context of enzymes that are associated with diet and metabolism, genes encoding for CAZymes; involved in the breakdown of complex carbohydrates [47] identified in the pre-Inca/Inca and Italian nobility mummies were further compared to those of modern Amazonians and European gut metagenomes. Identification of CAZyme sequences may be used to infer, to a certain extent, ancient dietary habits along with microbial identification, and archeological evidence. Analysis of shotgun metagenomics data revealed that the Italian nobility mummies had, on average, the highest number of CAZymes, although this difference did not meet statistical significance (Supplementary Figure S6A). CAZymes were included those with auxiliary activities (AA) (Supplementary Figure S6B), carbohydrate binding modules (CBM) (Supplementary Figure S6C), carbohydrate esterases (CE) (Supplementary Figure S6D), glycoside hydrolases (GH) (Supplementary Figure S6E), glycosyl transferases (GT) (Supplementary Figure S6F), and polysaccharide lyases (PL) (Supplementary Figure S6G). ANOVA results showed statistically significant differences for AA enzymes (*p* = 0.001), CBM (*p* < 0.001), CE (*p* = 0.011) and GT (*p* = 0.017) between the pre-Inca/Inca and Italian nobility mummies. No other statistically significant differences were noted. 

**Figure 4:**
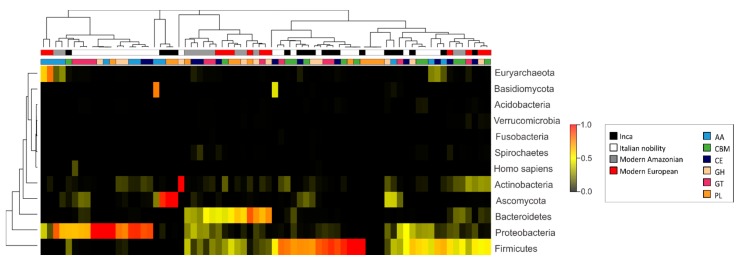
Carbohydrate-active enzymes (CAZyme) gene diversity in the pre-Inca/Inca and Italian nobility mummies guts. Contigs were uploaded to http://www.bioenergycenter.org/besc/index.cfm and annotated using a minimum identity of 80% and e-value of 10 × 10^−5^. The results represent the Euclidean distance of CAZyme gene counts for a taxon and CAZyme family. Taxon categories were manually curated and classified at the phylum level. Results were also compared to modern Amazonians and modern Europeans.

CAZymes are encoded by the microbiota; thus, identifying the presumptive microorganisms harboring CAZyme genes in mummified gut remains may provide insights into their similarities with CAZyme sequences that are present in modern human gut microbiomes, and how they might have potentially been preserved during the process of mummification. The most predominant taxa contributing CAZyme sequences differed between the mummified human gut remains and modern subjects. For instance, the Ascomycota (76.7%) and Firmicutes (14.26%) contributed the largest number of CAZyme genes in the pre-Inca/Inca gut metagenomes, whereas the Proteobacteria (55.3%) and Actinobacteria (41.2%) contributed the largest number of CAZyme genes in the Italian nobility gut metagenomes. Both the modern Amazonians and Europeans had the Firmicutes (25.7% and 40.1%, respectively), Proteobacteria (13.84% and 4.9%, respectively), Actinobacteria (5.6% and 23.6%, respectively), and Bacteroidetes (50.3% and 24.8%, respectively) contributing the largest number of CAZyme genes. Interestingly, Bacteroidetes (0.3% and 0.1%, respectively) contributing CAZyme genes were virtually non-existent in the mummified human gut remains, supporting the lack of detection of Bacteroidetes by 16S rRNA gene high throughput sequencing (Supplementary Figure S7). Generally, all of the identified microbial taxa contributed differently to the diversity of CAZyme genes that were identified in the mummified and modern human gut metagenomes, and no separation of the data was found according to group or enzyme type (Figure 4). For instance, the Firmicutes and Ascomycota contributed an average of approximately 77.6% of enzymes with AA in the pre-Inca/Inca mummies, whereas Proteobacteria contributed an average of approximately 65.4% of enzymes with AA in the Italian nobility mummies. The Firmicutes contributed 48.9%, 19.0%, 20.6%, and 33.9% of the GH in the pre-Inca/Inca, Italian nobility, modern Amazonians and modern Italians, respectively. The Proteobacteria accounted for 20.0%, 52.0%, 9.9%, and 2.4% of the GH in the pre-Inca/Inca, Italian nobility, modern Amazonians and modern Italians, respectively. The Firmicutes contributed 69.7%, 16.6%, 18.1%, and 25.7% of GT in the pre-Inca/Inca, Italian nobility, modern Amazonians, and modern Italians, respectively. The Proteobacteria accounted for 19.5%, 58.8%, 22.6%, and 8.5% of the GT in the pre-Inca/Inca, Italian nobility, modern Amazonians, and modern Italians, respectively (Figure 4). 

### 3.5. Putative Gut Resistomes of Pre-Inca/Inca and Italian Nobility Mummies

**Figure 5:**
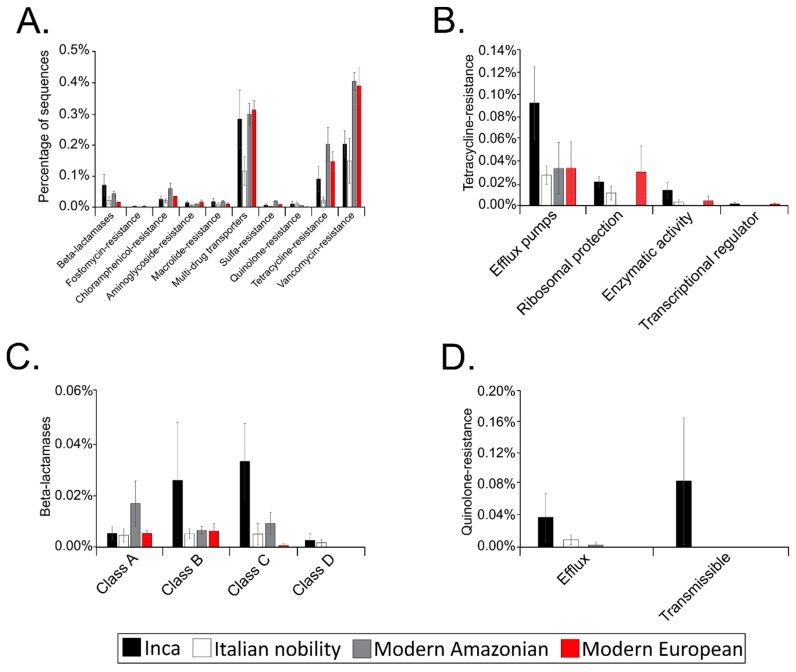
Putative resistome profiles of the pre-Inca/Inca and Italian nobility mummies. (**A**) Percentage of sequences similar to genes encoding for putative β-lactamases, multidrug transporters and resistance to fosfomycin, chloramphenicol, aminoglycoside, macrolide, sulfa, quinolone, tetracycline and vancomycin; (**B**) Putative tetracycline resistome showing the percentage of sequences similar to genes encoding for efflux pumps, ribosomal protection proteins, proteins with enzymatic activity and transcriptional regulators; (**C**) Putative β-lactamase resistome showing the percentage of sequences similar to genes encoding for Class A, Class B, Class C, and Class D β-lactamases; (**D**) Putative quinolone resistome showing the percentage of sequences similar to genes encoding for efflux pumps and those that are transmissible. Results were also compared to modern Amazonians and modern Europeans.

The putative gut resistomes of the pre-Inca/Inca and Italian nobility mummies were compared to those of modern Amazonians and Europeans to understand putative antibiotic-resistance patterns that could potentially provide insights of a gut resistome prior the antibiotic therapy era. Resistome refers to all of the antibiotic-resistance genes present in both pathogens and commensal microorganisms. The pre-Inca/Inca (3.29%) had a higher proportion of the contigs that were assigned to putative antibiotic-resistance genes when compared to the Italian nobility (1.58%); yet, this proportion was less compared to modern Amazonian (4.96%), and modern Europeans (3.65%). Only the percentage of putative antibiotic-resistance genes identified in the Italian nobility mummies and modern Amazonians met statistical significance (ANOVA; *p* = 0.015). Sequences that were associated with putative vancomycin-resistance and multidrug transporters were the most abundant in both the ancient and modern cultures (Figure 5A). Other putative antibiotic-resistance genes that are present in the ancient and modern cultures included fosfomycin-, chloramphenicol-, aminoglycoside-, macrolide-, sulfa-, quinolone-, and tetracycline-resistance (Figure 5A). Statistically significant differences were noted between several of the groups depending on the putative antibiotic-resistance category. For instance, ANOVA results showed that putative chloramphenicol- (*p* = 0.035), sulfa- (*p* < 0.001), and tetracycline-resistance (*p* = 0.001) genes were significantly different between the Italian nobility and modern Amazonians. ANOVA results also showed that putative multidrug transporters (*p* = 0.046) and tetracycline-resistance (*p* = 0.001) genes were significantly different between the Italian nobility and modern Europeans. Putative sulfa-resistance genes were significantly different between modern Amazonians and Europeans (*p* = 0.013). No other significant differences were noted. 

Selected putative antibiotic-resistance gene categories, including tetracycline-resistance, β-lactamases and quinolone-resistance were further categorized based on the mechanism of action. Tetracycline-resistance sequences were further characterized in the present study because of its origin in the environment [48]. Similarly, β-lactamases have also been shown to have originated in the environment and date to thousands of years [49]. Identification of quinolone-resistance sequences sharing similarity to modern quinolone-resistance genes is intriguing as quinolones are synthetic drugs [50]. ANOVA results showed that the relative abundance of efflux pumps in the tetracycline-resistance category was significantly different between the pre-Inca/Inca (0.091%) and the Italian nobility (0.026%) mummies (*p* = 0.031) (Figure 5B). No other significant differences were noted in the putative tetracycline-resistance category. The relative abundance of class C in the β-lactamases category was significantly different between the pre-Inca/Inca (0.003%) and Italian nobility mummies (0.005%) (*p* = 0.038). No other significant differences were noted in the putative β-lactamases category (Figure 5C). While the highest proportion of putative quinolone-resistance genes were present in the pre-Inca/Inca culture and were categorized as transmissible (0.038%), no statistically significant differences were noted between the groups (Figure 5D).

**Figure 6:**
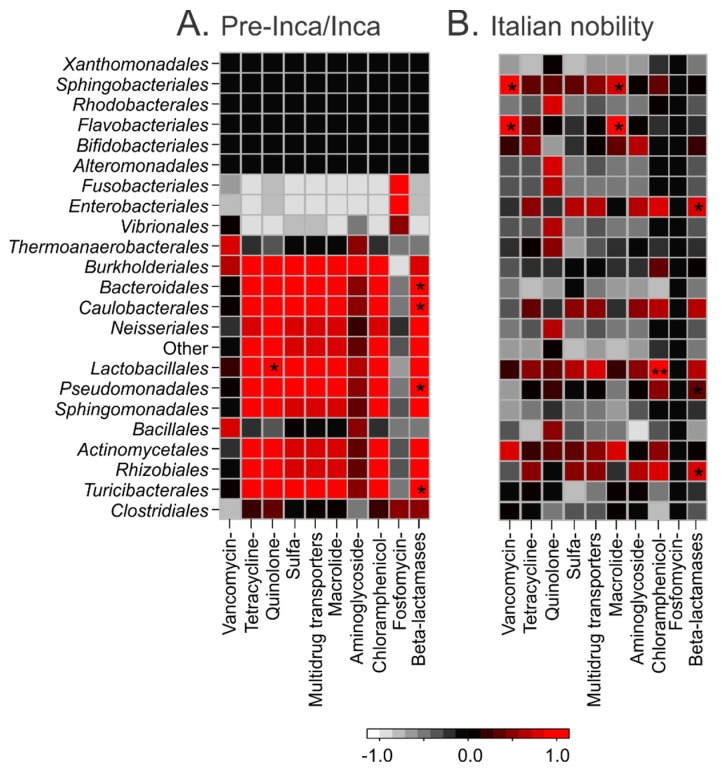
Pearson’s correlations between taxonomy at the order level (left) and the resistome (bottom) of the pre-Inca/Inca (Panel **A**) and Italian nobility (Panel **B**) mummies. Positive correlations are shown in red, negative correlations are shown in white and no correlations are shown in black. Significance is represented by asterisks: *p* ≤ 0.05 (*) and *p* ≤ (0.01) (**).

Pairwise correlations of the relative abundances of the taxa and the putative resistome categories were performed to explore potential associations. Pairwise statistically significant correlations (*p* ≤ 0.01) of putative β-lactamases and quinolone-resistance genes with taxonomic assignments in the pre-Inca/Inca mummies are shown with an asterisk (*) (Figure 6A). No other significant correlations were noted in the pre-Inca/Inca mummies. Pairwise statistically significant correlations (*p* ≤ 0.01) of putative β-lactamases, macrolide-, and vancomycin-resistance with taxonomic assignments in the Italian nobility mummies are shown with an asterisk (*). Pairwise statistically significant correlations (*p* ≤ 0.001) of chloramphenicol-resistance with taxonomic assignments are shown with asterisks (**) (Figure 6B). No other significant correlations were noted in the Italian nobility mummies. 

## 4. Discussion

The present study adds knowledge of the process of natural and artificial mummification of the human gut by characterizing the preserved microbiome of five Italian nobility mummies in comparison with three previously characterized pre-Inca/Inca mummies. While the very diverse characteristics of the mummies, including age, gender, morbidities, pathologies, lifestyles, and dietary habits may challenge associations made with sequence information, it is evident that the process of natural and artificial mummification of the human gut preserves bacterial and fungal DNA, as well as genes associated with metabolism and putative antibiotic-resistance genes. Our study took advantage of not only microbial identification, but also of genes associated with metabolism to infer dietary habits and lifestyles, which also support the archeological observations. 

It is challenging to authenticate ancient human microbiomes using damage-based tools that were developed for the authentication of ancient human DNA [3]. In the present study, mapDamage results did not show the peculiar damage patterns that are associated with ancient DNA [38]. Typical sequence patterns resulting from ancient DNA damage include short sequence length, an excess of cytosine to thymine (C-to-T) misincorporations at 5′ ends, and complementary guanine to adenine (G-to-A) misincorporations at the 3′ ends [51]. Damage-based ancient DNA authentication tools, such as mapDamage, may be incompatible with ancient microbiome studies unless a high sequencing coverage is reached [51]. For instance, the mapDamage output from modern human, Otzi’s and *Treponema denticola* DNA demonstrated differing outcomes [52]. Interestingly, DNA damage in *T. denticola* was not restricted to the 5′ end, and the damage patterns occurred at an order of magnitude higher when compared to Otzi’s DNA. The possibility remains that the absence of mismatches directly at the 5′ end may be a technical artifact resulting from the mapping tool utilized in the present study. In addition, no modern microbiomes contributed to the findings that are reported in the present study based on the SourceTracker results. Notably, the microbiomes used as the “source” were modern gut and oral microbiomes from USA individuals. This may account for the lack of resemblance of the majority of the mummies with modern gut microbiomes [53]. While the results should be carefully interpreted in ancient microbiome studies, SourceTracker can potentially be used to identify and filter sequences that could represent contaminating sources of DNA. Contamination with modern DNA would have also been noted in the agarose gels, with high-molecular weight bands, which was not the case, as the DNA was highly fragmented [54]. Reporting sequences present in non-template or blank controls is becoming essential for data interpretation in microbiome research. Studies may report data prior and after filtering sequences identified in non-template or blank controls; however, it has also been suggested that filtering sequences found in non-template or blank controls may not increase the resolution of the lineages as some may authentically be present in microbiome samples [55].

The process of natural and artificial mummification of the human gut may reflect both the thanatomicrobiome and the original human gut microbiome, and may be representative of the various forms of the process of mummification that was practiced by pre-Inca/Inca and Italian nobility cultures. For instance, it is known that the gut thanatomicrobiome is characterized by a higher proportion of *Clostridiales*, and a reduction of *Lactobacillales* and *Bacteroidales* [56]. The mummies included in the present study exhibited a significant abundance of *Clostridiales* (as shown with both 16S rRNA gene high-throughput sequencing and shotgun metagenomics data), and the presence of other taxa that are usually associated with a healthy human gut microbiome, including members of the *Enterobacteriales* and *Bifidobacteriales* [57]. The higher bacterial alpha diversity using 16S rRNA high-throughput sequencing in the Italian nobility mummies is intriguing as it suggests that differences in dietary habits (plant- vs. animal-based) may support a previous study that an animal-based affects bacterial diversity of the human gut [58]. The majority of the sequences can usually be classified at the phylum level, but classification using ≥97% identity can become challenging at higher levels of classification [59]. Discrepancies between 16S rRNA gene and shotgun metagenomic data are also an issue that has been noted, and which is not restricted to the analysis of the dataset in the present study. It has been shown that more taxa can be identified when analyzing shotgun metagenomic data, and has been suggested that extracting 16S rRNA gene sequences from shotgun metagenomic datasets may eliminate biases introduced by 16S rRNA gene PCR amplification [60]. 

From the shotgun metagenomics data it is evident that the process of natural and artificial mummification of the human gut preserves microbial DNA. Several of the groups that were identified in the mummified gut remains, including the *Clostridiales*, *Bifidobacteriales*, *Enterobacteriales*, *Bacillales*, and *Lactobacillales* are known to be indigenous members of the gut of modern humans [61]. The *Enterobacteriales* being a unique feature of the pre-Inca/Inca mummies guts opens the opportunity to investigate which members of this diverse group are preserved during the process of natural and artificial mummification. Similarly, the *Leptospirales* were unique features of the pre-Inca-Inca mummies, supporting that the pre-Inca/Incas were in direct contact with the environment. *Leptospirales* can colonize various environments, and are also symbionts of different animal hosts. Members of this order are also known to be human pathogens [58]. It also needs to be further investigated if the differences noted in bacterial relative abundances are due to the true differences that are associated with the process of natural and/or artificial mummification, dietary habits, and health and disease. While, only the relative abundances of bacteria were presented, further analyses will investigate the fungal and viral composition of the mummies included in the present study. 

Shotgun metagenomics in microbiome research has been primarily utilized to understand changes in microbial community structure and function; yet, shotgun metagenomics may also have the potential of identifying plant and animal sequences, which may be used to reconstruct dietary habits of ancient cultures. A similar approach was applied to characterize dietary habits of apes and archaeobotanical remains [62,63], and more recently, Neanderthal dietary habits and behavior [25]; yet, no studies have applied a similar approach to infer, to a certain extent, dietary habits from mummified gut remains. While archeological and historical records have provided insights into the dietary habits of the pre-Inca/Inca and Italian nobility, the present study took advantage of results from shotgun metagenome sequencing to add to this knowledge. Given that shotgun metagenomics is becoming more accessible and there is an increasing number of updated and curated plant and animal sequences, a similar approach can be used to determine the dietary habits of diverse ancient cultures, particularly those with no direct archeological or historical records. 

Genes that are associated with specific types of metabolism may also provide insights into ancient dietary habits. Recently, microbial sequence data found in Neanderthal dental calculi have been used to infer dietary habits and behavior [25]. While data should be carefully interpreted, the advantage of analyzing genes that are associated with specific types of metabolism is that they might provide more direct evidence of ancient dietary habits than microbial, animal and plant species identification alone. Among the main types of metabolism, namely carbohydrate, amino acid and lipid metabolism, more is known about the enzymatic diversity involved in carbohydrate metabolism, also known as CAZymes [64,65]. A greater average proportion of CAZyme genes were found in both the pre-Inca/Inca and Italian nobility mummies as compared to modern Amazonians and Italians. The high number of CAZyme genes in the Italian nobility may have been driven by the higher number of CAZyme genes in mummies NASD3 (Pietro of Aragona) and NASD22 (Ferrante I of Aragona). Interestingly, mummy NASD3 was the only child, and NASD22 was the only artificial mummy included in the present study. While CAZyme gene identification should be carefully utilized to infer dietary habits, the higher number of CAZyme sequences in the Italian nobility mummies may suggest that they had a higher capacity to degrade complex carbohydrates when compared to the Incas, modern Amazonians and Italians; yet, the higher proportion of AA enzymes in the Incas is intriguing as it suggests a greater ligninolytic and lytic polysaccharide mono-oxygenase activities [66]. Results may also suggest that CAZyme sequences are preserved in differing degrees during the process of natural or artificial mummification of the human gut. 

The human gut microbiome is also known to harbor a robust resistome [67]. With the increasing number of curated and updated databases, such as the Comprehensive Antibiotic Resistance Database (CARD), antibiotic-resistance profiles, and evolution can be addressed from high-throughput sequencing data. While antibiotic-resistance is more associated with antibiotic therapy, an increasing number of studies have demonstrated that the human gut microbiome of isolated cultures also possess a robust resistome [65]. The gut resistome of the pre-Inca/Inca and Italian nobility mummies lends further evidence of the human gut as a reservoir of antibiotic-resistance genes prior the antibiotic therapy era. The higher proportions of putative β-lactamases, multidrug transporters, and tetracycline-resistance genes in the pre-Inca/Inca mummies and modern Amazonian, when compared to the Italian nobility is in agreement with the hypothesis that a lifestyle with a greater exposure to the environment and consumption of soil-contaminated crops may result in a higher acquisition of antibiotic-resistance genes [68]. Antibiotic-resistance is hypothesized to have an environmental origin as antibiotic production results from the secondary metabolism of diverse microorganisms. These microorganisms must have co-evolved resistance in order to avoid auto-toxicity. Transmission of antibiotic-resistance genes to other commensal microorganisms remains a matter of further research and speculation. In addition, it has been repeatedly suggested that antibiotic resistance in pathogens likely had their origins in non-pathogenic bacteria, possibly those originating from soil. It has also been hypothesized that resistance to more recent antibiotics possibly emerged from resistance to earlier forms of antibiotics. This may explain the similarity percentages <97% of the putative antibiotic–resistance genes identified in the mummies when compared to modern antibiotic-resistance genes. The putative resistomes of the mummified gut remains exhibited a number of complex associations diverse taxa. These interactions could potentially be used to infer distinct dietary habits, lifestyles and evolution.

## Data Availability

16S data for the Inca mummies are available in MG-RAST under ID numbers 4644220.3 (descending colon mummy FI9), 4662510.3 (mummy FI3) and 4662511.3 (mummy FI12). 16S data for the Italian mummies are available in MGRAST under ID numbers 4769343.3 (mummy NASD3), 4769345.3 (mummy NASD14), 4769342.3 (mummy NASD22), 4769341.3 (mummy NASD27) and 4769344.3 (mummy NASD29). Shotgun metagenomic data are available in MGRAST under ID numbers 4630170.3 (descending colon mummy FI9), 4629033.3 (mummy FI3), and 4626489.3 (mummy FI12). Shotgun metagenomic data for the Italian mummies are available in MGRAST under ID numbers 4629038.3 (mummy NASD3), 4629034.3 (mummy NASD14), 4629035.3 (mummy NASD22), 4629036.3 (mummy NASD27), and 4629037.3 (mummy NASD29).

## Supplementary Materials

The following are available online at www.mdpi.com/2073-4425/8/11/310/s1.

## References

[1] Adler C.J., Dobney K., Weyrich L.S., Kaidonis J., Walker A.W., Haak W., Bradshaw C.J., Townsend G., Soltysiak A., Alt K.W. (2013). Sequencing ancient calcified dental plaque shows changes in oral microbiota with dietary shifts of the Neolithic and Industrial revolutions. Nat. Genet..

[2] Hunter P. (2014). Pulling teeth from history: DNA from ancient teeth can help to yield information about our ancestors’ health, diet and diseases. EMBO Rep..

[3] Warinner C., Speller C., Collins M.J. (2015). A new era in palaeomicrobiology: Prospects for ancient dental calculus as a long-term record of the human oral microbiome. Philos. Trans. R. Soc. Lond. B Biol. Sci..

[4] Cano R.J., Rivera-Perez J., Toranzos G.A., Santiago-Rodriguez T.M., Narganes-Storde Y.M., Chanlatte-Baik L., Garcia-Roldan E., Bunkley-Williams L., Massey S.E. (2014). Paleomicrobiology: Revealing fecal microbiomes of ancient indigenous cultures. PLoS ONE.

[5] Santiago-Rodriguez T.M., Narganes-Storde Y.M., Chanlatte L., Crespo-Torres E., Toranzos G.A., Jimenez-Flores R., Hamrick A., Cano R.J. (2013). Microbial communities in pre-Columbian coprolites. PLoS ONE.

[6] Appelt S., Armougom F., le Bailly M., Robert C., Drancourt M. (2014). Polyphasic analysis of a middle ages coprolite microbiota, Belgium. PLoS ONE.

[7] Appelt S., Fancello L., le Bailly M., Raoult D., Drancourt M., Desnues C. (2014). Viruses in a 14th-century coprolite. Appl. Environ. Microbiol..

[8] Luciani S., Fornaciari G., Rickards O., Labarga C.M., Rollo F. (2006). Molecular characterization of a pre-Columbian mummy and in situ coprolite. Am. J. Phys. Anthropol..

[9] Janaway R.C., Wilson A.C., Carpio-Díaz G., Guillen S. (2009). Taphonomic Changes to the Buried Body in Arid Environments: An Experimental Case Study in Peru.

[10] Aufderheide A.C. (2003). The Scientific Study of Mummies.

[11] Vreeland J.M. (1998). Mummies of Peru. Mummies, Disease, and Ancient Cultures.

[12] Fornaciari A., Giuffra V., Pezzini F. (2010). Secondary burial and mummification practices in the Kingdom of the two Sicilies. Mortality.

[13] Fornaciari G. (2006). The Aragonese mummies of the Basilica of Saint domenico maggiore in Naples. Med. Secoli.

[14] Ubaldi M., Luciani S., Marota I., Fornaciari G., Cano R.J., Rollo F. (1998). Sequence analysis of bacterial DNA in the colon of an Andean mummy. Am. J. Phys. Anthropol..

[15] Tito R.Y., Knights D., Metcalf J., Obregon-Tito A.J., Cleeland L., Najar F., Roe B., Reinhard K., Sobolik K., Belknap S. (2012). Insights from characterizing extinct human gut microbiomes. PLoS ONE.

[16] Santiago-Rodriguez T.M., Fornaciari G., Luciani S., Dowd S.E., Toranzos G.A., Marota I., Cano R.J. (2015). Gut Microbiome of an 11th Century AD Pre-Columbian Andean Mummy. PLoS ONE.

[17] Santiago-Rodriguez T.M., Fornaciari G., Luciani S., Dowd S.E., Toranzos G.A., Marota I., Cano R.J. (2016). Taxonomic and predicted metabolic profiles of the human gut microbiome in pre-Columbian mummies. FEMS Microbiol. Ecol..

[18] Santiago-Rodriguez T.M., Fornaciari G., Luciani S., Dowd S.E., Toranzos G.A., Marota I., Cano R.J. (2016). Natural mummification of the human gut preserves bacteriophage DNA. FEMS Microbiol. Lett..

[19] Piperno D.R., Dillehay T.D. (2008). Starch grains on human teeth reveal early broad crop diet in northern Peru. Proc. Natl. Acad. Sci. USA.

[20] Livengood S.V. (2012). Refining Dietary Estimates at Machu Picchu Using Combined Dental Macro/Microwear and Isotopic Analyses. Master’s Thesis.

[21] Malpass M.A. (2009). Daily Life in the Inca Empire.

[22] Fornaciari G. (2008). Food and disease at the Renaissance courts of Naples and Florence: A paleonutritional study. Appetite.

[23] Riera-Melis A., Flandrin J.L., Montanari M. (1999). Society, food and Feudalism. Food: A Culinary History from Antiquity to the Present.

[24] Grieco A.J., Flandrin J.L., Montanari M. (1999). Food and social classes in late Medieval and Renaissance Italy. Food: A Culinary History from Antiquity to the Present.

[25] Weyrich L.S., Duchene S., Soubrier J., Arriola L., Llamas B., Breen J., Morris A.G., Alt K.W., Caramelli D., Dresely V. (2017). Neanderthal behaviour, diet, and disease inferred from ancient DNA in dental calculus. Nature.

[26] Fornaciari G., Castagna M., Viacava P., Tognetti A., Bevilacqua G., Segura E.L. (1992). Chagas’ disease in Peruvian Inca mummy. Lancet.

[27] Fornaciari G. (1985). The mummies of the Abbey of Saint Domenico Maggiore in Naples: A preliminary report. Arch. Antrop. Etnol..

[28] Fornaciari G., Pollina L., Tornabuoni D., Tognetti A. (1988). Pulmonary and hepatic pathologies in the series of mummies of S. Domenico Maggiore at Naples (XVI century). Advances in Paleopathology: Proceeding of the VII European meeting of Paleopathology Association, Lyon, September 1988.

[29] Gaeta R., Ventura L., Fornaciari G. (2015). Il tumore di Ferdinando Orsini, duca di Gravina di Puglia (+1549). Atti del 50° Congrsso Nazionale della Società Italiana di Storia della Medicina.

[30] Gaeta R., Ventura L., Fornaciari G. (2017). The Cutaneous Cancer of Ferdinando Orsini, 5th Duke of Gravina. Jama Dermatol..

[31] Gaeta R., Giuffra V., Fornaciari G. (2017). Cancer in the Renaissance court of Naples. Lancet Oncol..

[32] Fornaciari G. (2017). Histology of ancient soft tissue tumors: A review. Int. J. Paleopathol..

[33] Marchetti A., Pellegrini S., Bevilacqua G., Fornaciari G. (1996). K-RAS mutation in the tumour of Ferrante I of Aragon, King of Naples. Lancet.

[34] Ottini L., Falchetti M., Marinozzi S., Angeletti L.R., Fornaciari G. (2011). Gene-environment interactions in the pre–Industrial Era: The cancer of King Ferrante I of Aragon (1431–1494). Hum. Pathol..

[35] Ciranni R., Fornaciari G. (2004). Juvenile cirrhosis in a 16th century Italian mummy. Current technologies in pathology and ancient human tissues. Virchows Archiv..

[36] Yang B., Wang Y., Qian P.Y. (2016). Sensitivity and correlation of hypervariable regions in 16S rRNA genes in phylogenetic analysis. BMC Bioinform..

[37] Rideout J.R., He Y., Navas-Molina J.A., Walters W.A., Ursell L.K., Gibbons S.M., Chase J., McDonald D., Gonzalez A., Robbins-Pianka A. (2014). Subsampled open-reference clustering creates consistent, comprehensive OTU definitions and scales to billions of sequences. PeerJ.

[38] Ginolhac A., Rasmussen M., Gilbert M.T., Willerslev E., Orlando L. (2011). mapDamage: Testing for damage patterns in ancient DNA sequences. Bioinformatics.

[39] Pati A., Heath L.S., Kyrpides N.C., Ivanova N. (2011). ClaMS: A Classifier for Metagenomic Sequences. Stand. Geno. Sci..

[40] Sharpton T.J. (2014). An introduction to the analysis of shotgun metagenomic data. Front. Plant Sci..

[41] Meyer F., Paarmann D., D’Souza M., Olson R., Glass E.M., Kubal M., Paczian T., Rodriguez A., Stevens R., Wilke A. (2008). The metagenomics RAST server—A public resource for the automatic phylogenetic and functional analysis of metagenomes. BMC Bioinform..

[42] Knights D., Kuczynski J., Charlson E.S., Zaneveld J., Mozer M.C., Collman R.G., Bushman F.D., Knight R., Kelley S.T. (2011). Bayesian community-wide culture-independent microbial source tracking. Nat. Methods.

[43] Browne H.P., Forster S.C., Anonye B.O., Kumar N., Neville B.A., Stares M.D., Goulding D., Lawley T.D. (2016). Culturing of ‘unculturable’human microbiota reveals novel taxa and extensive sporulation. Nature.

[44] Stearns J.C., Lynch M.D., Senadheera D.B., Tenenbaum H.C., Goldberg M.B., Cvitkovitch D.G., Croitoru K., Moreno-Hagelsieb G., Neufeld J.D. (2011). Bacterial biogeography of the human digestive tract. Sci. Rep..

[45] Menguer P.K., Sperotto R.A., Ricachenevsky F.K. (2017). A walk on the wild side: Oryza species as source for rice abiotic stress tolerance. Genet. Mol. Biol..

[46] Sanchez E., Quesada T., Espinoza A.M. (2006). Ultrastructure of the wild rice *Oryza grandiglumis* (Gramineae) in Costa Rica. Rev. Biol. Trop..

[47] El Kaoutari A., Armougom F., Leroy Q., Vialettes B., Million M., Raoult D., Henrissat B. (2013). Development and validation of a microarray for the investigation of the CAZymes encoded by the human gut microbiome. PLoS ONE.

[48] Thaker M., Spanogiannopoulos P., Wright G.D. (2010). The tetracycline resistome. Cell. Mol. Life Sci..

[49] D’Costa V.M., King C.E., Kalan L., Morar M., Sung W.W.L., Schwarz C., Froese D., Zazula G., Calmels F., Debruyne R. (2011). Antibiotic resistance is ancient. Nature.

[50] Tran J.H., Jacoby G.A. (2002). Mechanism of plasmid-mediated quinolone resistance. Proc. Natl. Acad. Sci. USA.

[51] Ziesemer K.A., Mann A.E., Sankaranarayanan K., Schroeder H., Ozga A.T., Brandt B.W., Zaura E., Waters-Rist A., Hoogland M., Salazar-Garcia D.C. (2015). Intrinsic challenges in ancient microbiome reconstruction using 16S rRNA gene amplification. Sci. Rep..

[52] Maixner F., Thomma A., Cipollini G., Widder S., Rattei T., Zink A. (2014). Metagenomic analysis reveals presence of *Treponema denticola* in a tissue biopsy of the Iceman. PLoS ONE.

[53] Yatsunenko T., Rey F.E., Manary M.J., Trehan I., Dominguez-Bello M.G., Contreras M., Magris M., Hidalgo G., Baldassano R.N., Anokhin A.P. (2012). Human gut microbiome viewed across age and geography. Nature.

[54] Pääbo S., Poinar H., Serre D., Jaenicke-Després V., Hebler J., Rohland N., Kuch M., Krause J., Vigilant L., Hofreiter M. (2004). Genetic analyses from ancient DNA. Annu. Rev. Genet..

[55] Kim D., Hofstaedter C.E., Zhao C., Mattei L., Tanes C., Clarke E., Lauder A., Sherrill-Mix S., Chehoud C., Kelsen J. (2017). Optimizing methods and dodging pitfalls in microbiome research. Microbiome.

[56] DeBruyn J.M., Hauther K.A. (2017). Postmortem succession of gut microbial communities in human cadavers. PeerJ.

[57] Shreiner A.B., Kao J.Y., Young V.B. (2015). The gut microbiome in health and in disease. Curr. Opin. Gastroenterol..

[58] David L.A., Maurice C.F., Carmody R.N., Gootenberg D.B., Button J.E., Wolfe B.E., Ling A.V., Devlin A.S., Varma Y., Fischbach M.A. (2014). Diet rapidly and reproducibly alters the human gut microbiome. Nature.

[59] Nguyen N.-P., Warnow T., Pop M., White B. (2016). A perspective on 16S rRNA operational taxonomic unit clustering using sequence similarity. npj Biofilms Microb..

[60] Poretsky R., Rodriguez-R L.M., Luo C., Tsementzi D., Konstantinidis K.T. (2014). Strengths and limitations of 16S rRNA gene amplicon sequencing in revealing temporal microbial community dynamics. PLoS ONE.

[61] Ramadan M., Solyman S., Taha M., Hanora A. (2016). Preliminary characterization of human skin microbiome in healthy Egyptian individuals. Cell Mol. Biol..

[62] Hamad I., Delaporte E., Raoult D., Bittar F. (2014). Detection of termites and other insects consumed by African great apes using molecular fecal analysis. Sci. Rep..

[63] Nistelberger H.M., Smith O., Wales N., Star B., Boessenkool S. (2016). The efficacy of high-throughput sequencing and target enrichment on charred archaeobotanical remains. Sci. Rep..

[64] Tasse L., Bercovici J., Pizzut-Serin S., Robe P., Tap J., Klopp C., Cantarel B.L., Coutinho P.M., Henrissat B., Leclerc M. (2010). Functional metagenomics to mine the human gut microbiome for dietary fiber catabolic enzymes. Genome Res..

[65] Rampelli S., Schnorr S.L., Consolandi C., Turroni S., Severgnini M., Peano C., Brigidi P., Crittenden A.N., Henry A.G., Candela M. (2015). Metagenome Sequencing of the Hadza Hunter-Gatherer Gut Microbiota. Curr. Biol..

[66] Chen Y.R., Sarkanen S., Wang Y.Y. (2012). Lignin-degrading enzyme activities. Methods Mol. Biol..

[67] Hu Y., Yang X., Qin J., Lu N., Cheng G., Wu N., Pan Y., Li J., Zhu L., Wang X. (2013). Metagenome-wide analysis of antibiotic resistance genes in a large cohort of human gut microbiota. Nat. Commun..

[68] Allen H.K., Moe L.A., Rodbumrer J., Gaarder A., Handelsman J. (2009). Functional metagenomics reveals diverse β-lactamases in a remote Alaskan soil. ISME J..

