# Supplementary material for: Gut Microbiome and Putative Resistome of Inca and Italian Nobility Mummies
Source: Genes (Basel). 2017 Nov 7;8(11):310. doi: 10.3390/genes8110310 (PMC5704223; doi:10.3390/genes8110310)
Supplement: Supplementary file 1 [file genes-08-00310-s001.zip › Supplementary Table 1-2.docx]

**Supplementary Table 1.** Sample description

| **Mummy** | **Identity** | **Period or Family** | **Title** | **Century** | **Gender** | **Approximate age of death** | **Morbidities and pathologies** |
| --- | --- | --- | --- | --- | --- | --- | --- |
| FI9 | - | Pre-Inca | - | 10-11th | Female | 18 | Chagas' disease. Possible leishmaniasis. HPV infection |
| FI3 | - | Inca | - | 14-15th | Male | 25 | None |
| FI12 | - | Inca | - | 15-16th | Female | 20 | Bronchopneumonia |
| NASD3 | Pietro d’Aragona | Renaissance Naples | III Duke of Montalto | 16th | Male | 12 | Bronchopneumonia |
| NASD14 | Ferdinando Orsini | Renaissance Naples | V Duke of Gravina | 16th | Male | 50-55 | Facial skin carcinoma |
| NASD22* | Ferrante I d'Aragona | Renaissance Naples | King of Naples | 15th | Male | 70 | Colorectal carcinoma |
| NASD27 | Luigi Carafa | Renaissance Naples | II Prince of Stigliano | 16th | Male | 65 | - |
| NASD29 | Unknown | Renaissance Naples | Unknown | 16th | Unknown | 20-25 | Cirrhosis |

*Artificial mummy.
