# Supplementary material for: Gut Microbiome and Putative Resistome of Inca and Italian Nobility Mummies
Source: Genes (Basel). 2017 Nov 7;8(11):310. doi: 10.3390/genes8110310 (PMC5704223; doi:10.3390/genes8110310)
Supplement: Supplementary file 1 [file genes-08-00310-s001.zip › Supplementary Table 2_Shared OTUs.docx]

**Supplementary Table 2.** Shared OTUs (phylotypes) between the Pre-Inca/Inca and Italian nobility mummies.

|  | **Pre-Inca/Inca** | | |  | **Italian nobility** | | | | |
| --- | --- | --- | --- | --- | --- | --- | --- | --- | --- |
| **Mummy** | **FI9** | **FI3** | **FI12** |  | **NASD3** | **NASD14** | **NASD22** | **NASD27** | **NASD29** |
| **FI9** | 238 | 26 | 51 |  | 67 | 88 | 85 | 74 | 82 |
| **FI3** | 26 | 442 | 27 |  | 49 | 54 | 61 | 41 | 63 |
| **FI12** | 51 | 27 | 510 |  | 57 | 86 | 106 | 61 | 76 |
| **NASD3** | 67 | 49 | 57 |  | 719 | 501 | 421 | 385 | 351 |
| **NASD14** | 88 | 54 | 86 |  | 501 | 1058 | 602 | 437 | 494 |
| **NASD22** | 85 | 61 | 106 |  | 421 | 602 | 1346 | 383 | 581 |
| **NASD27** | 74 | 41 | 61 |  | 385 | 437 | 383 | 667 | 322 |
| **NASD29** | 82 | 63 | 76 |  | 351 | 494 | 581 | 322 | 1098 |
