# Supplementary material for: Gut Microbiome and Putative Resistome of Inca and Italian Nobility Mummies
Source: Genes (Basel). 2017 Nov 7;8(11):310. doi: 10.3390/genes8110310 (PMC5704223; doi:10.3390/genes8110310)
Supplement: Supplementary file 1 [file genes-08-00310-s001.zip › Supplementary Table 3.docx]

**Supplementary Table 3:** Relative abundance (%) of OTUs assigned at the genus in pre-Inca/Inca and Italian nobility mummies’ guts.

|  | **Pre-Inca/Inca** | | |  | **Italian nobility** | | | | |
| --- | --- | --- | --- | --- | --- | --- | --- | --- | --- |
| **Genus** | **FI3** | **FI9** | **FI12** |  | **NASD3** | **NASD14** | **NASD22** | **NASD27** | **NASD29** |
| Unclassified | 99.747% | 69.407% | 1.023% |  | 29.471% | 27.857% | 12.493% | 45.979% | 63.976% |
| Other | 0.013% | 0.485% | 0.109% |  | 2.574% | 0.447% | 20.818% | 0.702% | 0.123% |
| *Clostridium* | 0.022% | 28.410% | 69.711% |  | 0.527% | 10.455% | 1.961% | 3.739% | 1.549% |
| *Turicibacter* | 0.005% | 0.493% | 0.000% |  | 0.000% | 0.002% | 0.000% | 0.000% | 0.000% |
| *Cryocola* | 0.004% | 0.315% | 0.009% |  | 0.041% | 0.054% | 0.264% | 6.114% | 0.056% |
| *Sphingomonas* | 0.003% | 0.198% | 0.023% |  | 35.118% | 14.812% | 0.506% | 8.736% | 0.222% |
| *Pseudomonas* | 0.001% | 0.125% | 0.000% |  | 2.029% | 2.303% | 0.134% | 2.306% | 0.060% |
| *Aggregatibacter* | 0.000% | 0.077% | 0.000% |  | 0.000% | 0.023% | 0.002% | 0.000% | 0.001% |
| *Streptococcus* | 0.014% | 0.073% | 0.009% |  | 1.178% | 2.169% | 0.149% | 5.411% | 0.099% |
| *Bacillus* | 0.006% | 0.061% | 0.014% |  | 0.072% | 0.391% | 0.435% | 1.149% | 0.192% |
| *Tepidimicrobium* | 0.011% | 0.040% | 0.000% |  | 0.209% | 1.021% | 2.136% | 0.986% | 13.977% |
| *Corynebacterium* | 0.001% | 0.036% | 0.004% |  | 7.414% | 0.850% | 0.762% | 6.999% | 0.087% |
| *Veillonella* | 0.000% | 0.036% | 0.000% |  | 0.362% | 0.007% | 0.000% | 0.004% | 0.006% |
| *Methylobacterium* | 0.011% | 0.024% | 0.012% |  | 3.734% | 0.721% | 0.048% | 5.026% | 0.067% |
| *Bradyrhizobium* | 0.003% | 0.024% | 0.009% |  | 0.115% | 0.142% | 0.027% | 0.520% | 0.033% |
| *Paenibacillus* | 0.003% | 0.024% | 0.000% |  | 0.000% | 0.000% | 0.000% | 0.000% | 0.002% |
| *Acinetobacter* | 0.001% | 0.024% | 0.002% |  | 3.718% | 0.899% | 0.116% | 3.905% | 0.057% |
| *Anoxybacillus* | 0.000% | 0.024% | 0.000% |  | 0.000% | 0.000% | 0.000% | 0.000% | 0.000% |
| *Ammoniphilus* | 0.004% | 0.020% | 0.000% |  | 0.000% | 0.000% | 0.000% | 0.000% | 0.000% |
| *Neisseria* | 0.003% | 0.020% | 0.007% |  | 0.000% | 0.000% | 0.000% | 0.000% | 0.000% |
| *Thermoanaerobacterium* | 0.000% | 0.016% | 0.000% |  | 0.000% | 0.000% | 0.000% | 0.000% | 0.000% |
| *Rhodoplanes* | 0.000% | 0.012% | 0.000% |  | 0.000% | 0.014% | 0.000% | 0.004% | 0.004% |
| *Sporanaerobacter* | 0.001% | 0.008% | 28.990% |  | 0.324% | 0.549% | 26.663% | 2.821% | 1.441% |
| *Alkaliphilus* | 0.000% | 0.008% | 0.000% |  | 0.000% | 0.000% | 0.000% | 0.000% | 0.000% |
| *Brachybacterium* | 0.000% | 0.008% | 0.000% |  | 0.122% | 0.095% | 4.772% | 0.207% | 0.033% |
| *Brevibacterium* | 0.000% | 0.008% | 0.005% |  | 0.122% | 0.312% | 6.108% | 0.158% | 0.041% |
| *Prevotella* | 0.000% | 0.008% | 0.000% |  | 0.006% | 0.000% | 0.002% | 0.000% | 0.001% |
| *Virgibacillus* | 0.127% | 0.004% | 0.004% |  | 0.081% | 0.346% | 0.745% | 0.020% | 0.313% |
| *Delftia* | 0.000% | 0.004% | 0.000% |  | 0.988% | 0.289% | 0.022% | 0.057% | 0.018% |
| *Saccharopolyspora* | 0.000% | 0.004% | 0.028% |  | 0.178% | 0.066% | 10.625% | 0.479% | 0.041% |
| *Carnobacterium* | 0.005% | 0.000% | 0.000% |  | 0.000% | 0.000% | 0.000% | 0.000% | 0.000% |
| *Alloiococcus* | 0.003% | 0.000% | 0.000% |  | 0.577% | 0.016% | 0.027% | 0.012% | 0.016% |
| *Lactobacillus* | 0.003% | 0.000% | 0.002% |  | 0.371% | 0.007% | 0.017% | 0.008% | 0.016% |
| *Ralstonia* | 0.003% | 0.000% | 0.000% |  | 0.486% | 0.025% | 0.017% | 0.016% | 0.005% |
| *Vibrio* | 0.003% | 0.000% | 0.004% |  | 0.006% | 0.436% | 0.020% | 0.008% | 0.002% |
| *Fusobacterium* | 0.001% | 0.000% | 0.004% |  | 0.006% | 0.233% | 0.005% | 0.000% | 0.007% |
| *Photobacterium* | 0.001% | 0.000% | 0.000% |  | 0.056% | 4.140% | 0.065% | 0.028% | 0.019% |
| *Pseudonocardia* | 0.001% | 0.000% | 0.000% |  | 0.000% | 0.129% | 0.032% | 0.000% | 0.003% |
| *Selenomonas* | 0.001% | 0.000% | 0.000% |  | 0.486% | 0.005% | 0.003% | 0.211% | 0.006% |
| *Serratia* | 0.001% | 0.000% | 0.002% |  | 0.000% | 0.000% | 0.000% | 0.000% | 0.000% |
| *Achromobacter* | 0.000% | 0.000% | 0.000% |  | 0.000% | 0.000% | 0.007% | 0.000% | 0.000% |
| *Actinomyces* | 0.000% | 0.000% | 0.000% |  | 0.000% | 0.459% | 0.060% | 0.142% | 0.005% |
| *Actinomycetospora* | 0.000% | 0.000% | 0.000% |  | 0.000% | 0.000% | 0.003% | 0.000% | 0.000% |
| *Aequorivita* | 0.000% | 0.000% | 0.000% |  | 0.000% | 0.000% | 0.008% | 0.000% | 0.000% |
| *Aerococcus* | 0.000% | 0.000% | 0.000% |  | 0.000% | 0.002% | 0.015% | 0.000% | 0.002% |
| *Agrobacterium* | 0.000% | 0.000% | 0.000% |  | 0.012% | 0.090% | 0.005% | 0.004% | 0.003% |
| *Alcanivorax* | 0.000% | 0.000% | 0.000% |  | 0.393% | 0.034% | 0.041% | 0.016% | 0.003% |
| *Amycolatopsis* | 0.000% | 0.000% | 0.000% |  | 0.003% | 0.032% | 0.035% | 0.004% | 0.002% |
| *Anaerococcus* | 0.000% | 0.000% | 0.004% |  | 0.009% | 0.285% | 0.005% | 0.000% | 0.011% |
| *Arthrobacter* | 0.000% | 0.000% | 0.000% |  | 0.016% | 0.070% | 0.010% | 0.000% | 0.000% |
| *Atopobium* | 0.000% | 0.000% | 0.000% |  | 0.000% | 0.000% | 0.003% | 0.000% | 0.008% |
| *Bifidobacterium* | 0.000% | 0.000% | 0.000% |  | 0.003% | 0.000% | 0.002% | 0.004% | 0.000% |
| *Blastomonas* | 0.000% | 0.000% | 0.002% |  | 0.012% | 0.097% | 0.000% | 0.000% | 0.001% |
| *Brevibacillus* | 0.000% | 0.000% | 0.000% |  | 0.000% | 0.000% | 0.003% | 0.000% | 0.000% |
| *Brevundimonas* | 0.000% | 0.000% | 0.000% |  | 0.000% | 0.000% | 0.000% | 0.000% | 0.002% |
| *Brochothrix* | 0.000% | 0.000% | 0.000% |  | 0.009% | 0.567% | 0.028% | 0.004% | 0.031% |
| *Burkholderia* | 0.000% | 0.000% | 0.000% |  | 0.016% | 0.014% | 0.008% | 0.430% | 0.001% |
| *Caldicoprobacter* | 0.000% | 0.000% | 0.000% |  | 0.003% | 0.002% | 0.005% | 0.000% | 0.007% |
| *Candidatus Nitrososphaera* | 0.000% | 0.000% | 0.000% |  | 0.000% | 0.172% | 0.000% | 0.000% | 0.011% |
| *Catenibacterium* | 0.000% | 0.000% | 0.000% |  | 0.000% | 0.000% | 0.000% | 0.000% | 0.003% |
| *Cloacibacterium* | 0.000% | 0.000% | 0.000% |  | 0.003% | 0.005% | 0.007% | 0.004% | 0.002% |
| *Cohnella* | 0.000% | 0.000% | 0.000% |  | 0.000% | 0.000% | 0.000% | 0.000% | 0.004% |
| *Comamonas* | 0.000% | 0.000% | 0.000% |  | 0.876% | 0.009% | 0.007% | 0.008% | 0.006% |
| *Coprococcus* | 0.000% | 0.000% | 0.000% |  | 0.000% | 0.000% | 0.000% | 0.000% | 0.001% |
| *Curtobacterium* | 0.000% | 0.000% | 0.000% |  | 0.000% | 0.113% | 0.056% | 0.008% | 0.001% |
| *Cytophaga* | 0.000% | 0.000% | 0.000% |  | 0.003% | 0.097% | 0.002% | 0.000% | 0.001% |
| *Dechloromonas* | 0.000% | 0.000% | 0.000% |  | 0.000% | 0.011% | 0.000% | 0.000% | 0.000% |
| *Demequina* | 0.000% | 0.000% | 0.000% |  | 0.000% | 0.002% | 0.023% | 0.000% | 0.000% |
| *Dermacoccus* | 0.000% | 0.000% | 0.000% |  | 0.000% | 0.000% | 0.000% | 0.000% | 0.003% |
| *Desemzia* | 0.000% | 0.000% | 0.000% |  | 0.000% | 0.007% | 0.017% | 0.000% | 0.003% |
| *Dialister* | 0.000% | 0.000% | 0.000% |  | 0.000% | 0.000% | 0.000% | 0.000% | 0.003% |
| *Dietzia* | 0.000% | 0.000% | 0.000% |  | 0.003% | 0.000% | 0.015% | 0.000% | 0.000% |
| *Dorea* | 0.000% | 0.000% | 0.000% |  | 0.000% | 0.000% | 0.000% | 0.000% | 0.004% |
| *Enhydrobacter* | 0.000% | 0.000% | 0.000% |  | 0.022% | 0.407% | 0.013% | 0.016% | 0.008% |
| *Enterococcus* | 0.000% | 0.000% | 0.000% |  | 0.000% | 0.000% | 0.003% | 0.000% | 0.002% |
| *Epulopiscium* | 0.000% | 0.000% | 0.000% |  | 0.000% | 0.002% | 0.000% | 0.000% | 0.000% |
| *eromicrobium* | 0.000% | 0.000% | 0.000% |  | 0.000% | 0.197% | 0.013% | 0.004% | 0.000% |
| *Erysipelothrix* | 0.000% | 0.000% | 0.000% |  | 0.000% | 0.000% | 0.000% | 0.000% | 0.001% |
| *Filifactor* | 0.000% | 0.000% | 0.000% |  | 0.000% | 0.000% | 0.002% | 0.041% | 0.001% |
| *Finegoldia* | 0.000% | 0.000% | 0.000% |  | 0.894% | 0.023% | 0.022% | 0.004% | 0.032% |
| *Garciella* | 0.000% | 0.000% | 0.000% |  | 0.000% | 0.011% | 0.219% | 0.016% | 0.009% |
| *Georgenia* | 0.000% | 0.000% | 0.000% |  | 0.000% | 0.000% | 0.012% | 0.008% | 0.000% |
| *Gordonia* | 0.000% | 0.000% | 0.000% |  | 0.003% | 0.000% | 0.003% | 0.000% | 0.000% |
| *Granulicatella* | 0.000% | 0.000% | 0.000% |  | 0.000% | 0.000% | 0.000% | 0.000% | 0.001% |
| *Haemophilus* | 0.000% | 0.000% | 0.000% |  | 0.000% | 0.000% | 0.002% | 0.000% | 0.000% |
| *Halococcus* | 0.000% | 0.000% | 0.000% |  | 0.000% | 0.000% | 0.000% | 0.000% | 0.000% |
| *Halomonas* | 0.000% | 0.000% | 0.002% |  | 0.000% | 0.000% | 0.003% | 0.000% | 0.000% |
| *Janthinobacterium* | 0.000% | 0.000% | 0.000% |  | 0.006% | 0.025% | 0.003% | 0.191% | 0.003% |
| *Kocuria* | 0.000% | 0.000% | 0.000% |  | 0.003% | 0.000% | 0.005% | 0.000% | 0.000% |
| *Kribbella* | 0.000% | 0.000% | 0.000% |  | 0.009% | 0.000% | 0.012% | 0.341% | 0.001% |
| *Leptospirillum* | 0.000% | 0.000% | 0.000% |  | 0.000% | 0.000% | 0.000% | 0.004% | 0.001% |
| *Leptotrichia* | 0.000% | 0.000% | 0.000% |  | 0.000% | 0.002% | 0.002% | 0.000% | 0.000% |
| *Leucobacter* | 0.000% | 0.000% | 0.000% |  | 0.009% | 0.002% | 0.138% | 0.032% | 0.004% |
| *Leuconostoc* | 0.000% | 0.000% | 0.000% |  | 0.003% | 0.000% | 0.002% | 0.000% | 0.000% |
| *Lysinibacillus* | 0.000% | 0.000% | 0.000% |  | 0.003% | 0.000% | 0.002% | 0.000% | 0.002% |
| *Lysobacter* | 0.000% | 0.000% | 0.000% |  | 0.000% | 0.002% | 0.003% | 0.004% | 0.000% |
| *Mesorhizobium* | 0.000% | 0.000% | 0.004% |  | 0.000% | 0.000% | 0.000% | 0.000% | 0.000% |
| *Methylibium* | 0.000% | 0.000% | 0.000% |  | 0.016% | 0.020% | 0.008% | 0.345% | 0.003% |
| *Microbispora* | 0.000% | 0.000% | 0.000% |  | 0.841% | 0.002% | 0.017% | 0.016% | 0.001% |
| *Micrococcus* | 0.000% | 0.000% | 0.000% |  | 0.003% | 0.066% | 0.012% | 0.000% | 0.001% |
| *Mycobacterium* | 0.000% | 0.000% | 0.000% |  | 0.006% | 0.002% | 0.053% | 0.171% | 0.000% |
| *Mycoplana* | 0.000% | 0.000% | 0.000% |  | 0.041% | 0.023% | 0.007% | 0.848% | 0.009% |
| *Natronobacillus* | 0.000% | 0.000% | 0.000% |  | 0.411% | 0.707% | 1.191% | 0.309% | 16.315% |
| *Nitrospira* | 0.000% | 0.000% | 0.000% |  | 0.000% | 0.007% | 0.005% | 0.426% | 0.000% |
| *Oceanobacillus* | 0.000% | 0.000% | 0.000% |  | 0.000% | 0.000% | 0.002% | 0.000% | 0.001% |
| *Olivibacter* | 0.000% | 0.000% | 0.000% |  | 0.000% | 0.000% | 0.000% | 0.049% | 0.012% |
| *Parabacteroides* | 0.000% | 0.000% | 0.000% |  | 0.000% | 0.000% | 0.000% | 0.000% | 0.010% |
| *Paracoccus* | 0.000% | 0.000% | 0.000% |  | 0.000% | 0.000% | 0.003% | 0.000% | 0.000% |
| *Pedomicrobium* | 0.000% | 0.000% | 0.000% |  | 0.006% | 0.122% | 0.000% | 0.004% | 0.001% |
| *Peptoniphilus* | 0.000% | 0.000% | 0.000% |  | 0.003% | 0.221% | 0.003% | 0.012% | 0.010% |
| *Phenylobacterium* | 0.000% | 0.000% | 0.000% |  | 0.000% | 0.000% | 0.002% | 0.000% | 0.002% |
| *Pigmentiphaga* | 0.000% | 0.000% | 0.000% |  | 0.000% | 0.002% | 0.002% | 0.000% | 0.000% |
| *Pimelobacter* | 0.000% | 0.000% | 0.000% |  | 0.000% | 0.007% | 0.050% | 0.004% | 0.002% |
| *Planctomyces* | 0.000% | 0.000% | 0.000% |  | 0.000% | 0.000% | 0.000% | 0.000% | 0.000% |
| *Pontibacter* | 0.000% | 0.000% | 0.000% |  | 0.000% | 0.000% | 0.002% | 0.000% | 0.006% |
| *Prauserella* | 0.000% | 0.000% | 0.000% |  | 0.000% | 0.000% | 0.000% | 0.000% | 0.000% |
| *Propionibacterium* | 0.000% | 0.000% | 0.000% |  | 0.006% | 0.009% | 0.003% | 0.000% | 0.000% |
| *Providencia* | 0.000% | 0.000% | 0.000% |  | 0.000% | 0.002% | 0.002% | 0.000% | 0.000% |
| *Psychrobacter* | 0.000% | 0.000% | 0.000% |  | 0.056% | 0.972% | 0.032% | 0.057% | 0.008% |
| *Rheinheimera* | 0.000% | 0.000% | 0.000% |  | 0.009% | 0.395% | 0.012% | 0.016% | 0.009% |
| *Rickettsia* | 0.000% | 0.000% | 0.000% |  | 0.000% | 0.000% | 0.005% | 0.000% | 0.000% |
| *Roseateles* | 0.000% | 0.000% | 0.000% |  | 0.000% | 0.000% | 0.000% | 0.000% | 0.003% |
| *Rothia* | 0.000% | 0.000% | 0.000% |  | 0.000% | 0.000% | 0.000% | 0.000% | 0.000% |
| *Rubellimicrobium* | 0.000% | 0.000% | 0.000% |  | 0.003% | 0.002% | 0.000% | 0.004% | 0.006% |
| *Ruminococcus* | 0.000% | 0.000% | 0.000% |  | 0.000% | 0.000% | 0.002% | 0.000% | 0.000% |
| *Salinicoccus* | 0.000% | 0.000% | 0.000% |  | 0.000% | 0.005% | 0.007% | 0.000% | 0.001% |
| *Schwartzia* | 0.000% | 0.000% | 0.000% |  | 0.580% | 0.005% | 0.007% | 0.004% | 0.003% |
| *Segetibacter* | 0.000% | 0.000% | 0.004% |  | 0.000% | 0.000% | 0.000% | 0.000% | 0.000% |
| *SMB53* | 0.000% | 0.000% | 0.002% |  | 0.000% | 0.000% | 0.000% | 0.000% | 0.000% |
| *Sphingobacterium* | 0.000% | 0.000% | 0.000% |  | 0.000% | 0.007% | 0.164% | 0.000% | 0.003% |
| *Sphingobium* | 0.000% | 0.000% | 0.000% |  | 0.006% | 0.018% | 0.007% | 0.004% | 0.000% |
| *Sporosarcina* | 0.000% | 0.000% | 0.000% |  | 0.000% | 0.005% | 0.018% | 0.000% | 0.004% |
| *Staphylococcus* | 0.000% | 0.000% | 0.016% |  | 4.535% | 25.800% | 8.242% | 0.804% | 0.893% |
| *Streptomyces* | 0.000% | 0.000% | 0.000% |  | 0.000% | 0.023% | 0.013% | 0.000% | 0.001% |
| *Tepidimonas* | 0.000% | 0.000% | 0.000% |  | 0.003% | 0.000% | 0.003% | 0.000% | 0.000% |
| *Tissierella Soehngenia* | 0.000% | 0.000% | 0.000% |  | 0.000% | 0.005% | 0.002% | 0.000% | 0.033% |
| *Trabulsiella* | 0.000% | 0.000% | 0.000% |  | 0.003% | 0.002% | 0.000% | 0.000% | 0.000% |
| *Yaniella* | 0.000% | 0.000% | 0.000% |  | 0.785% | 0.020% | 0.334% | 0.032% | 0.010% |
