# Supplementary material for: Gut Microbiome and Putative Resistome of Inca and Italian Nobility Mummies
Source: Genes (Basel). 2017 Nov 7;8(11):310. doi: 10.3390/genes8110310 (PMC5704223; doi:10.3390/genes8110310)
Supplement: Supplementary file 1 [file genes-08-00310-s001.zip › Supplementary Table 4_Read and contig information.docx]

**Supplementary Table 4.** Read and contig information of the Pre-Inca/Inca (FI9, FI3 and FI12) and Italian nobility (NASD3, NASD14, NASD22, NASD27 and NASD29) mummies included.

| **Mummy** | **Number of reads** | **Average read length (bp)** | **Number of contigs** | **Average contig length (bp)** |
| --- | --- | --- | --- | --- |
| FI9 | 146,081,692 | 100 | 25,630 | 733 |
| FI3 | 16,805,260 | 100 | 126,181 | 289 |
| FI12 | 16,537,474 | 100 | 36.466 | 242 |
| NASD3 | 16,570,556 | 100 | 25,279 | 243 |
| NASD14 | 17,687,976 | 100 | 50,874 | 262 |
| NASD22 | 18,959,490 | 100 | 64,371 | 232 |
| NASD27 | 16,847,766 | 100 | 30,373 | 454 |
| NASD29 | 14,265,734 | 100 | 92,440 | 386 |
