# Supplementary figures and images for: Gut Microbiome and Putative Resistome of Inca and Italian Nobility Mummies
Source: Genes (Basel). 2017 Nov 7;8(11):310. doi: 10.3390/genes8110310 (PMC5704223; doi:10.3390/genes8110310)

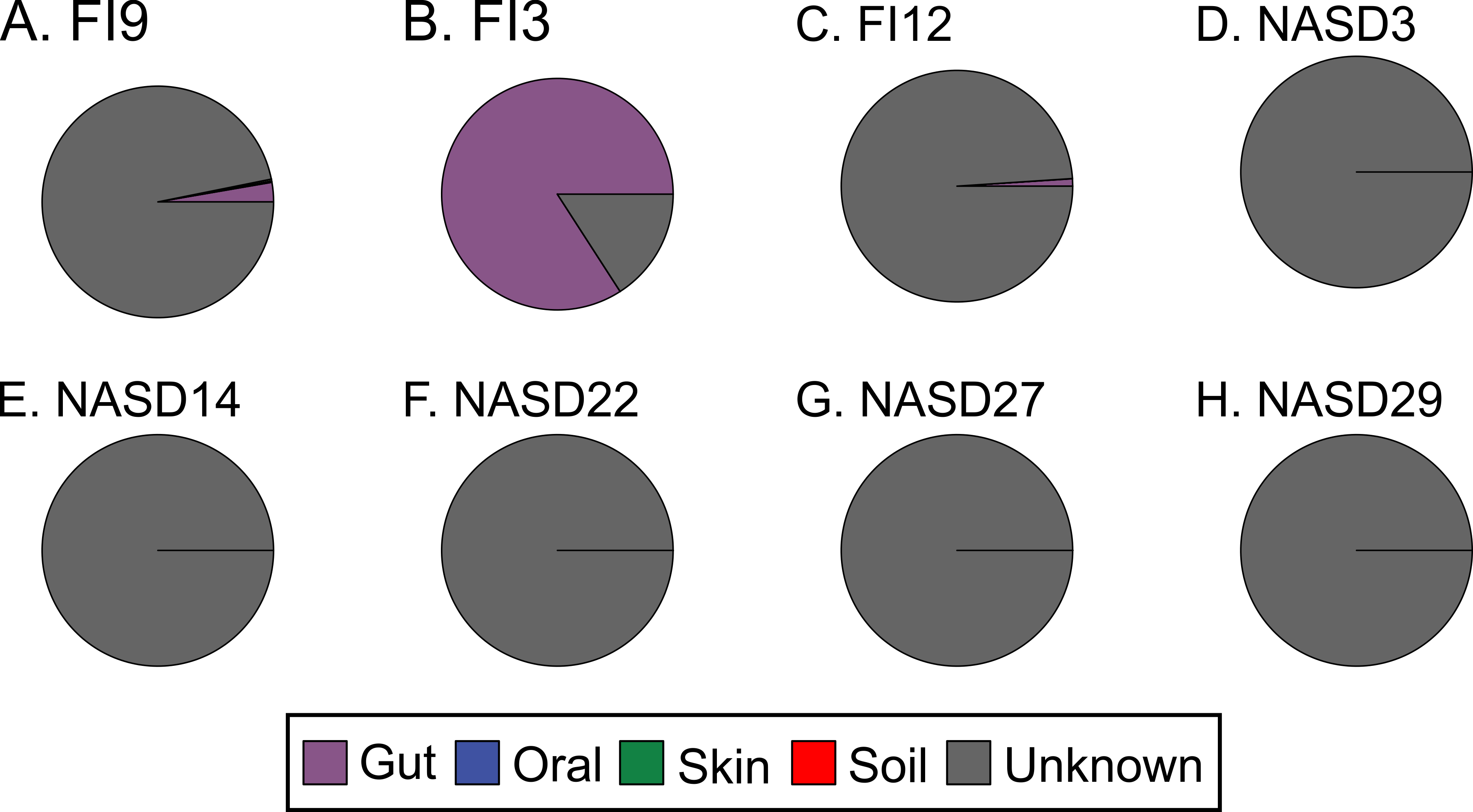

Supplement: Supplementary file 1 [file genes-08-00310-s001.zip › Supplementary Figure 1_Source_Tracker_All.tif]

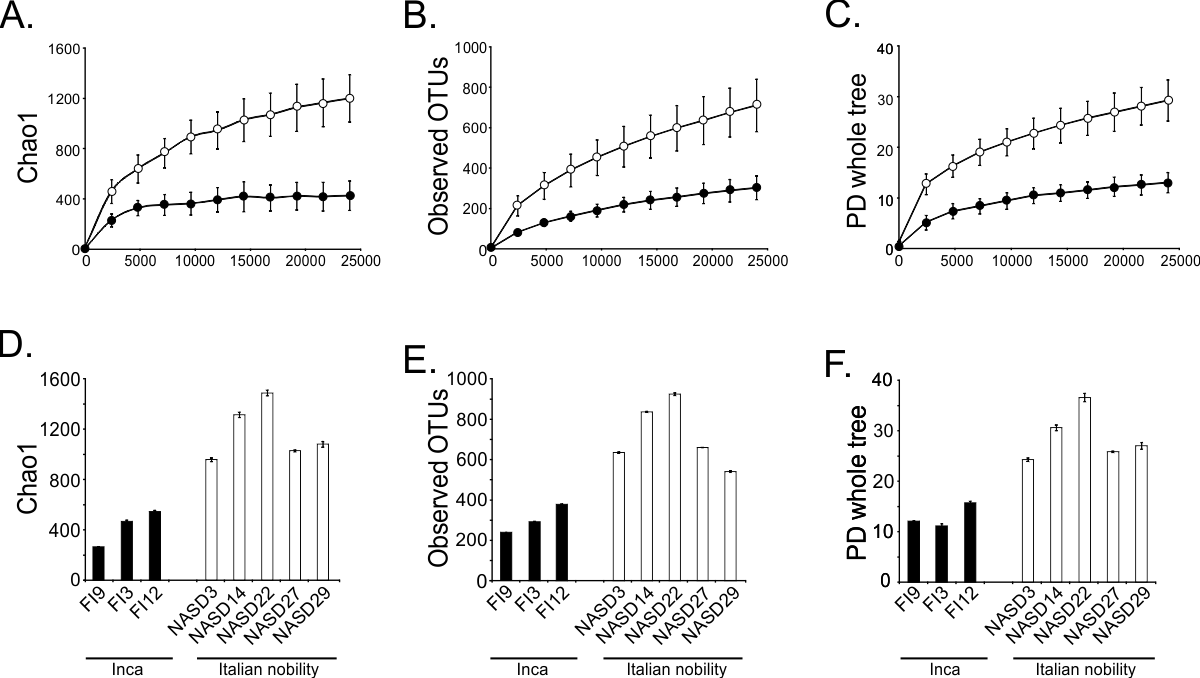

Supplement: Supplementary file 1 [file genes-08-00310-s001.zip › Supplementary Figure 2_Alpha diversities.tif]

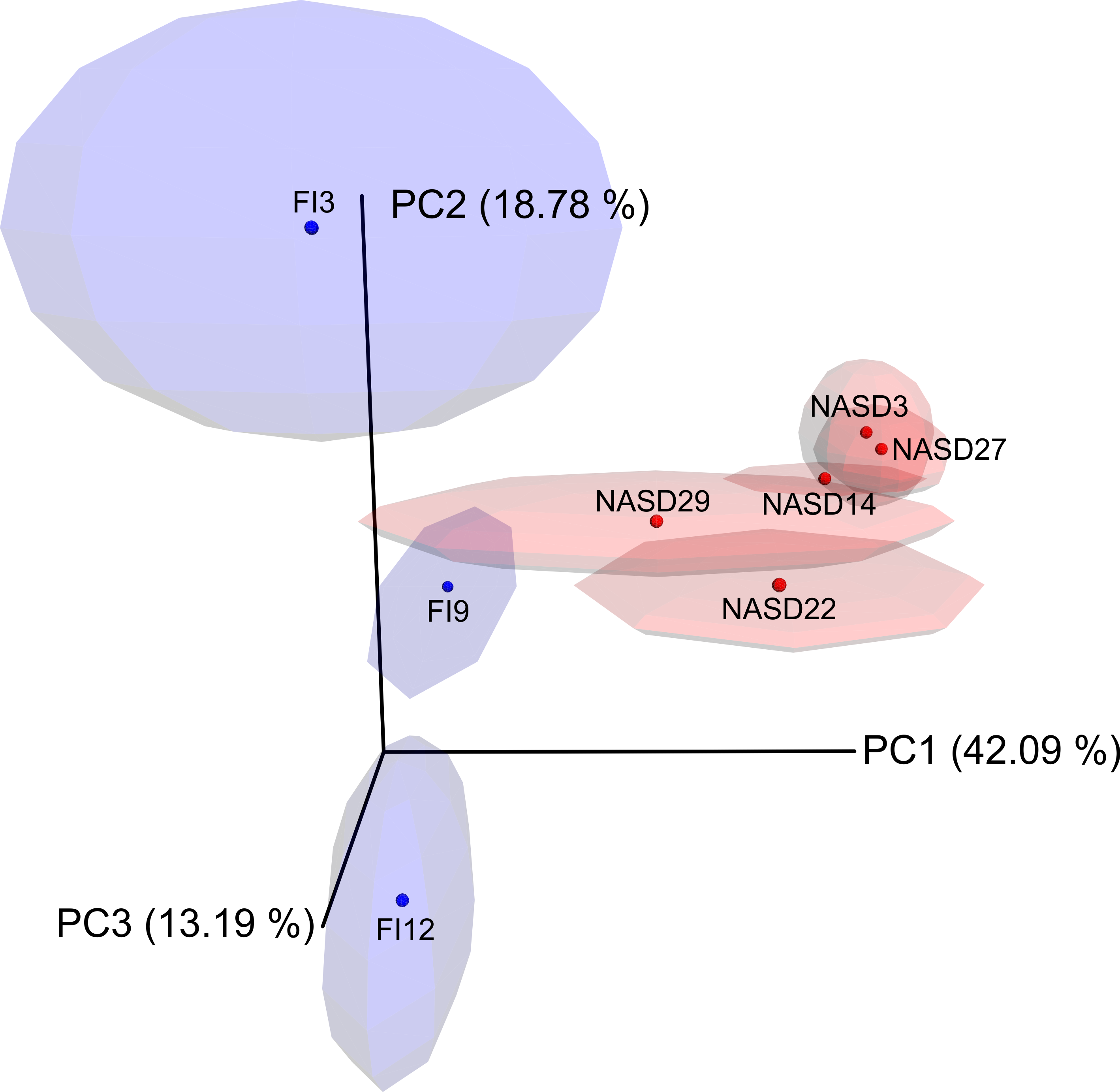

Supplement: Supplementary file 1 [file genes-08-00310-s001.zip › Supplementary Figure 3_Procrustes.tif]

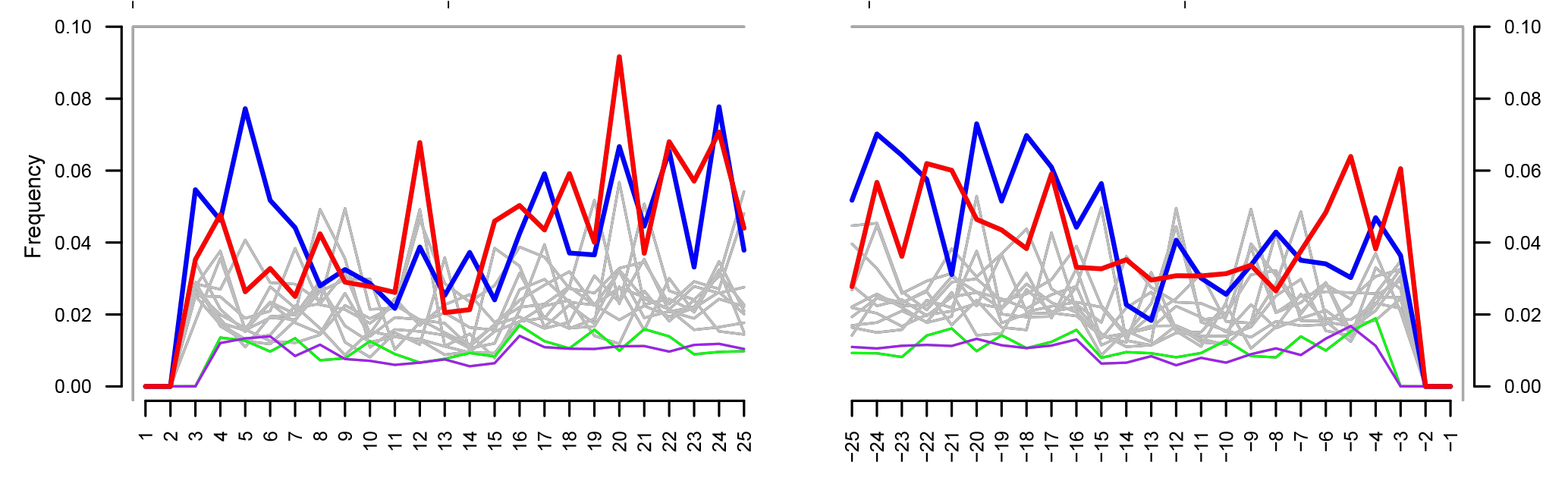

Supplement: Supplementary file 1 [file genes-08-00310-s001.zip › Supplementary Figure 4_MapDamage.tif]

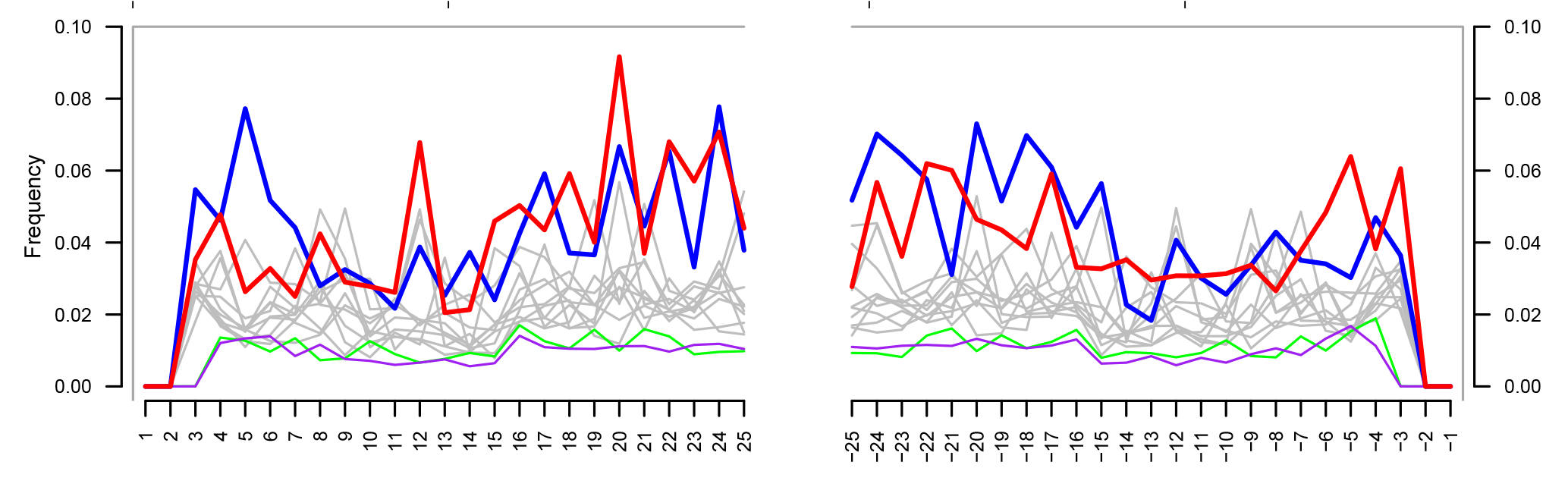

Supplement: Supplementary file 1 [file genes-08-00310-s001.zip › Supplementary Figure 4_MapDamage-2.tif]

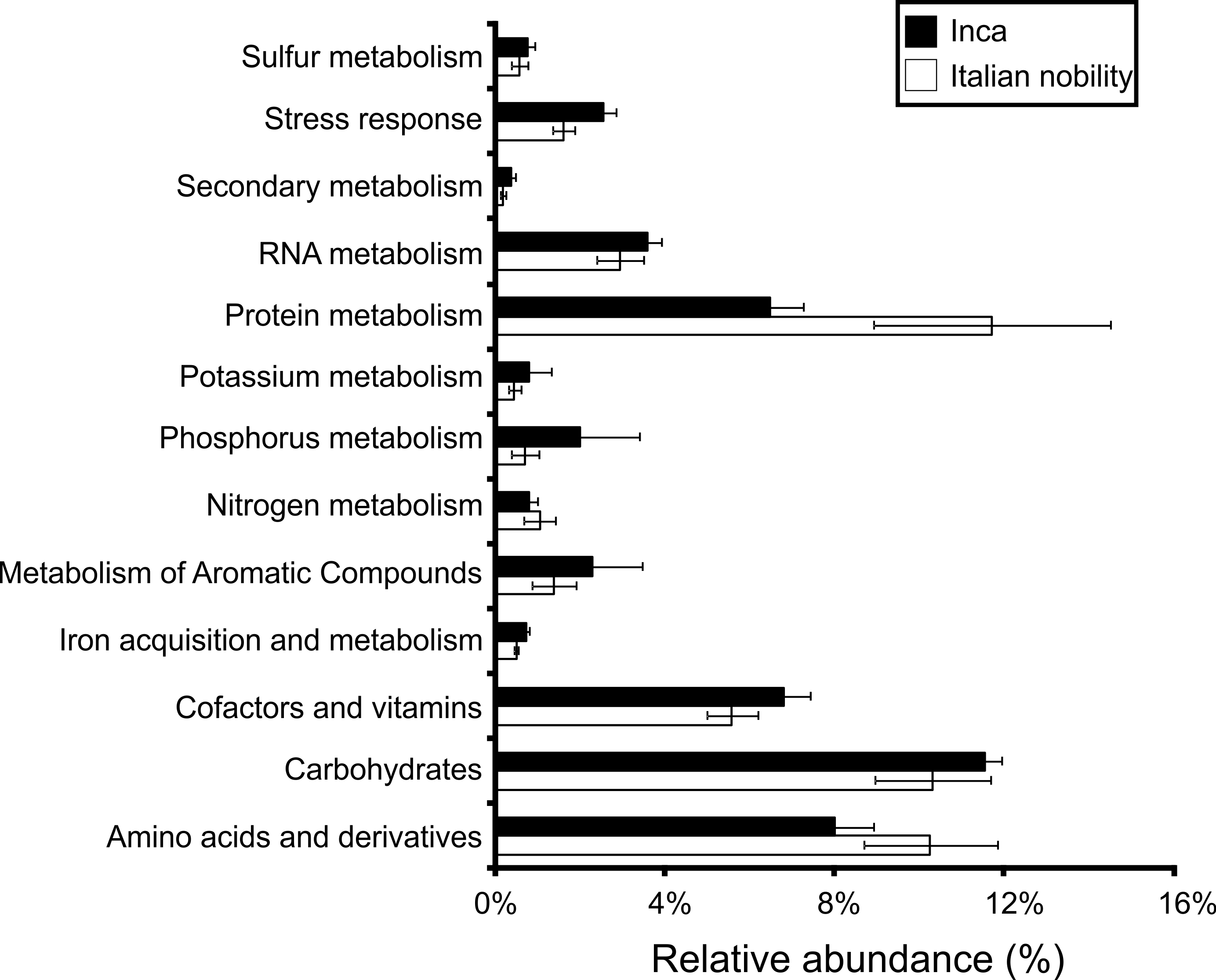

Supplement: Supplementary file 1 [file genes-08-00310-s001.zip › Supplementary Figure 5_Functional categories.tif]

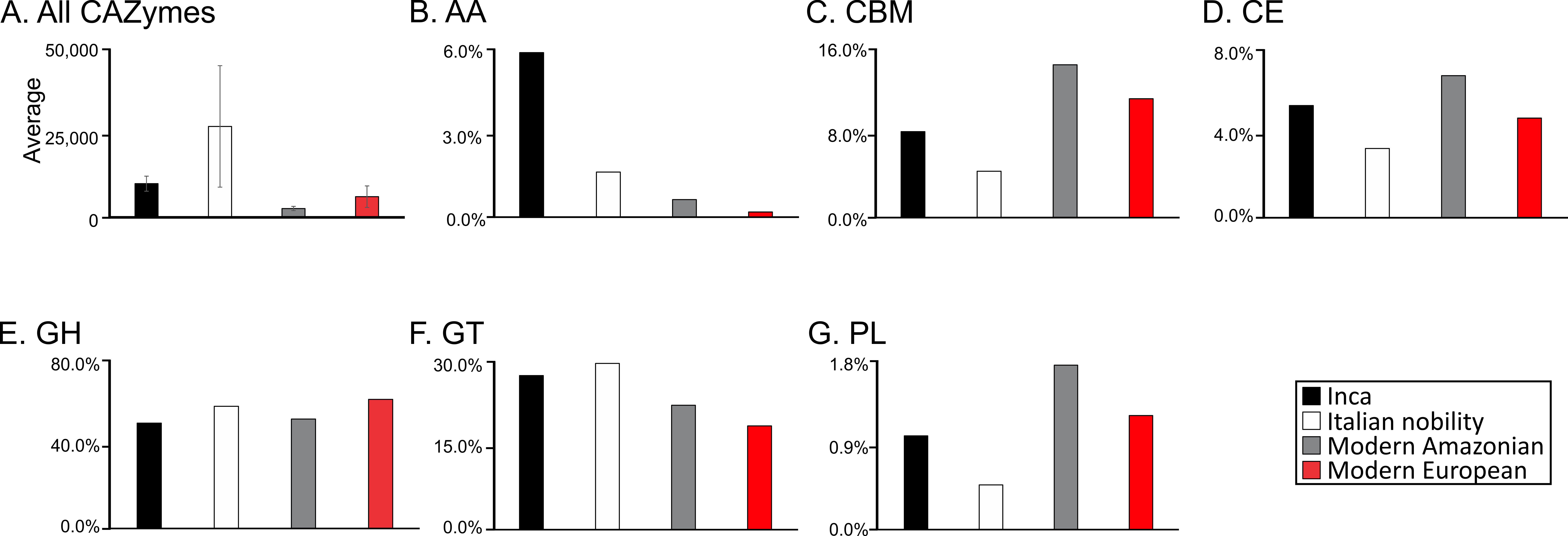

Supplement: Supplementary file 1 [file genes-08-00310-s001.zip › Supplementary Figure 6_CAZymes per type.tif]

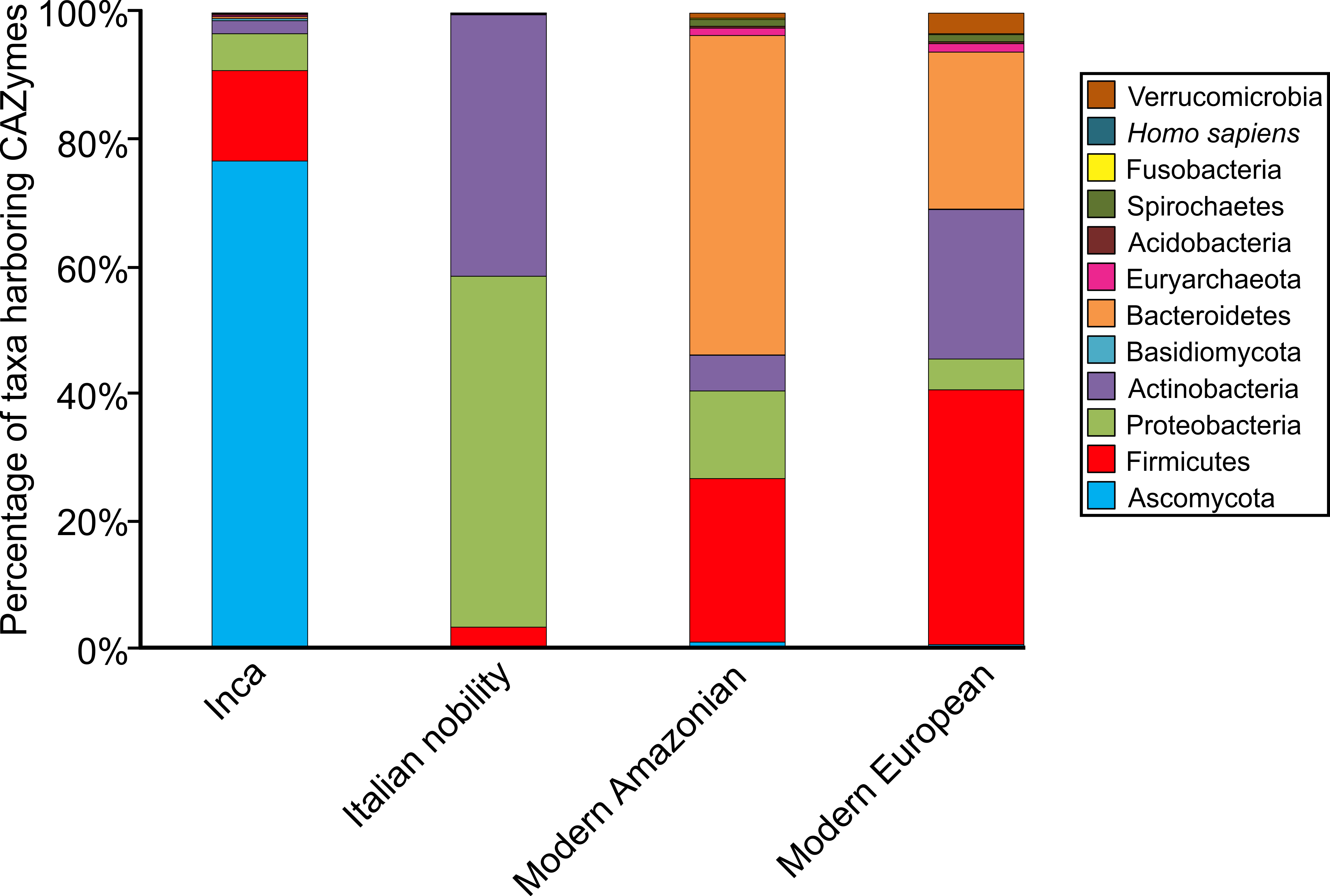

Supplement: Supplementary file 1 [file genes-08-00310-s001.zip › Supplementary Figure 7_Taxa contributing CAZymes.tif]
